# An extracellular [NiFe] hydrogenase mediating iron corrosion is encoded in a genetically unstable genomic island in *Methanococcus maripaludis*

**DOI:** 10.1038/s41598-018-33541-5

**Published:** 2018-10-11

**Authors:** Hirohito Tsurumaru, Naofumi Ito, Koji Mori, Satoshi Wakai, Taku Uchiyama, Takao Iino, Akira Hosoyama, Hanako Ataku, Keiko Nishijima, Miyako Mise, Ai Shimizu, Takeshi Harada, Hiroshi Horikawa, Natsuko Ichikawa, Tomohiro Sekigawa, Koji Jinno, Satoshi Tanikawa, Jun Yamazaki, Kazumi Sasaki, Syuji Yamazaki, Nobuyuki Fujita, Shigeaki Harayama

**Affiliations:** NITE Biological Resource Center (NBRC), National Institute of Technology and Evaluation (NITE), Chiba, 292-0818 Japan

## Abstract

Certain methanogens deteriorate steel surfaces through a process called microbiologically influenced corrosion (MIC). However, the mechanisms of MIC, whereby methanogens oxidize zerovalent iron (Fe^0^), are largely unknown. In this study, Fe^0^-corroding *Methanococcus maripaludis* strain OS7 and its derivative (strain OS7mut1) defective in Fe^0^-corroding activity were isolated. Genomic analysis of these strains demonstrated that the strain OS7mut1 contained a 12-kb chromosomal deletion. The deleted region, termed “MIC island”, encoded the genes for the large and small subunits of a [NiFe] hydrogenase, the TatA/TatC genes necessary for the secretion of the [NiFe] hydrogenase, and a gene for the hydrogenase maturation protease. Thus, the [NiFe] hydrogenase may be secreted outside the cytoplasmic membrane, where the [NiFe] hydrogenase can make direct contact with Fe^0^, and oxidize it, generating hydrogen gas: Fe^0^ + 2 H^+^ → Fe^2+^ + H_2_. Comparative analysis of extracellular and intracellular proteomes of strain OS7 supported this hypothesis. The identification of the MIC genes enables the development of molecular tools to monitor epidemiology, and to perform surveillance and risk assessment of MIC-inducing *M. maripaludis*.

## Introduction

The oxidation of zerovalent iron (Fe^0^) is accelerated by metabolic activities of certain microorganisms through a process called microbiologically influenced corrosion (MIC). Sulfate-reducing bacteria (SRB) are considered the main causal agents of MIC under anaerobic conditions through the formation of hydrogen sulfide, which is a very corrosive compound^1^. MIC induced by chemical reactions with biogenic compounds such as hydrogen sulfide is referred to as “chemical microbiologically influenced corrosion” or CMIC^2^.

The cathodic depolarization theory is another proposed mechanism of MIC, which has attracted both support and criticism. This theory assumes that the anaerobic oxidation of Fe^0^ (Fe^0^ ⇄ Fe^2+^ + 2e^−^; anodic reaction) coupled with the reduction of H^+^ (2 H^+^ + 2e^−^ ⇄ H_2_; cathodic reaction) is limited by the diffusion of H_2_ from the cathode, and that hydrogenotrophic microorganisms including SRB and methanogens accelerate the rate-limiting cathodic reaction by consuming H_2_ built up around Fe^0^ surfaces. However, the validity of this theory was questioned, as not all hydrogenotrohic SRB and methanogens promoted Fe^0^ corrosion^3,4^.

On the other hand, Zadvorny and colleagues^5^ found that hydrogenases from *Thiocapsa roseopersicina* and *Lamrobacter modestohalophilus* accelerated the corrosion of Fe^0^ and other metals with simultaneous evolution of H_2_. Based on the results, they proposed that the direct withdrawal of electrons from Fe^0^ by the hydrogenases accelerates Fe^0^ corrosion. Recently, Fe^0^ corrosion due to direct electron withdrawal has attracted much attention, and this mechanism is termed “electrical microbiologically influenced corrosion” or EMIC^2^. The direct electron transfer from an Fe^0^ electrode to cells of the Fe^0^-corroding SRB, *Desulfopila corrodens* IS4^3^, was experimentally demonstrated^6^. Further electrochemical and infrared spectroelectrochemical analyses indicated that the direct electron transfer from Fe^0^ to cells of strain IS4 occurred via *c*-type cytochromes located on the cell surface^7^. *c*-type cytochromes have been found in the outer membranes of particular microorganisms, which have the ability to acquire energy through transfer of electrons to extracellular electron acceptors or from extracellular electron donors^8^. The direct electron transfer from Fe^0^ to the cells of another SRB, *Desulfovibrio ferrophilus* IS5^3^, was also demonstrated^9^.

Methanogenic archaea may be second only to SRB as the most common cause of MIC in anaerobic environments^3,10–12^. Recently, Deutzmann *et al*.^13^ demonstrated that cell-free filtrates of spent cultures of the Fe^0^-corroding methanogen, *Methanococcus maripaludis* MM901, accelerated H_2_ generation from Fe^0^, while filtrates of spent cultures of strain MM1284, which carried mutations in five hydrogenase genes and one dehydrogenase gene, did not accelerate it. Since the activity in the filtrate of strain MM901 was thermolabile and proteinase-sensitive, they concluded that enzyme(s), most probably hydrogenase(s) secreted from strain MM901, catalyzed the electron withdrawal from Fe^0^.

Previously, we isolated and characterized Fe^0^-corroding *M. maripaludis* KA1^11^. In this study, we isolated another Fe^0^-corroding *M. maripaludis* strain, OS7, from which an Fe^0^-non-corroding mutant was isolated and characterized.

## Results and Discussion

### Isolation of Fe^0^-corroding *M. maripaludis* strain OS7 and its Fe^0^-non-corroding derivative, strain OS7mut1

An Fe^0^-corroding methanogen named strain OS7 was isolated from water sitting at the bottom of a crude-oil storage tank at Osaka by the method described previously for the isolation of Fe^0^-corroding *M. maripaludis* strain KA1^11^. The 16S rRNA gene sequence of strain OS7 was 100% identical to the 16S rRNA gene sequence of the type strain of *M. maripaludis* (strain JJ^T^); thus, strain OS7 was identified as belonging to the species *M. maripaludis*.

**Figure 1:**
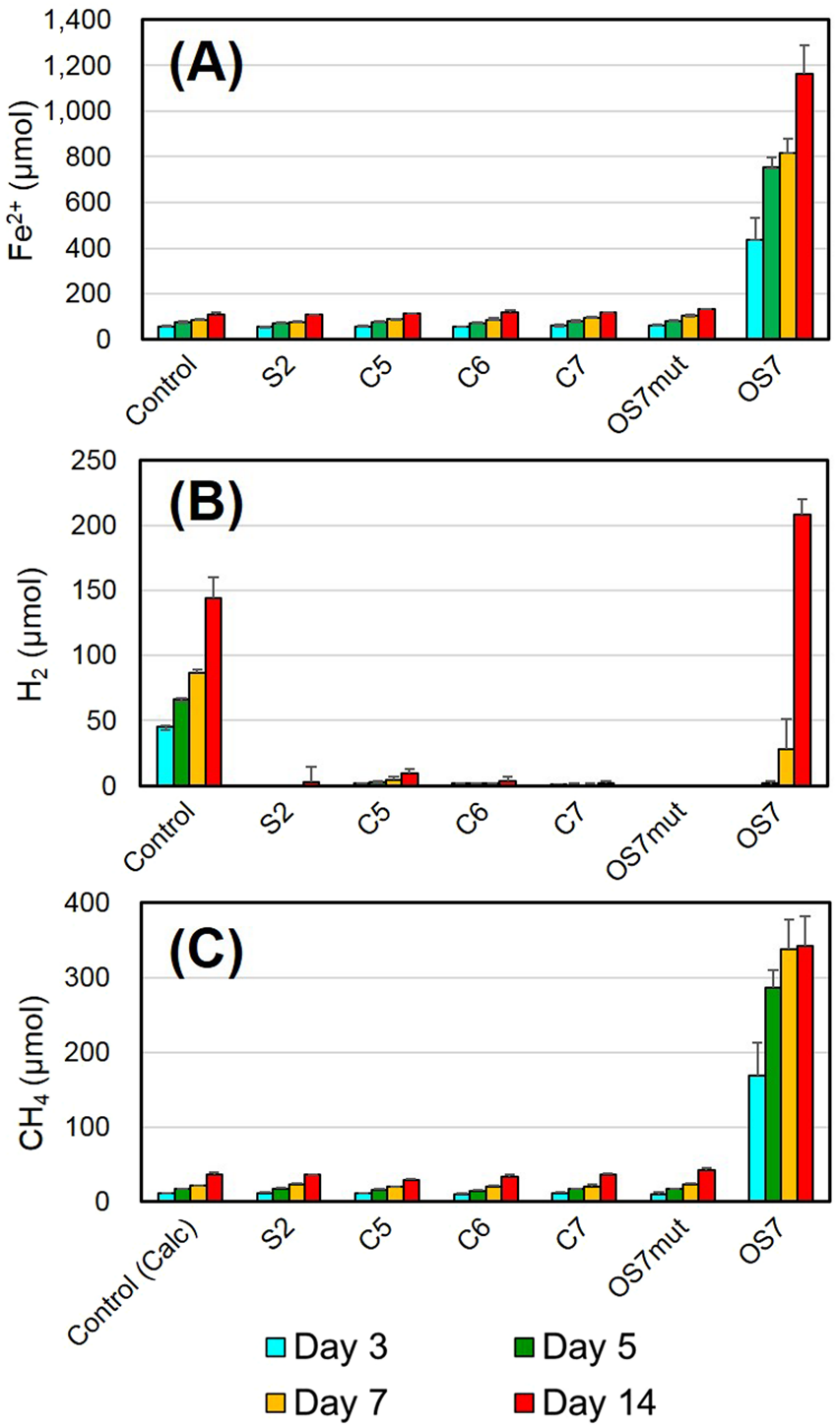
Fe^0^-corroding activities in various *M. maripaludis* strains. Amounts of Fe^2+^ in culture fluids **(A)**, amounts of H_2_ in headspace gas **(B)**, and amounts of CH_4_ in headspace gas **(C)** were determined after growth of the indicated strains for 3 to 14 days. Means and standard deviations of four determinations are shown. CH_4_ was not detected in headspace gas of the control samples, but amounts of CH_4_ which can be synthesized from H_2_ generated in the control samples are shown as “Control (Calc)”.

Similar to strain KA1, strain OS7 corroded Fe^0^ with concomitant CH_4_ production (Fig. 1). The amount of Fe^2+^ dissolved in a culture of strain OS7 at day 7 (816 μmol from 20 ml culture) was almost 10-fold higher than that produced in a sterile control (86 μmol from 20 ml culture). On the other hand, *M. maripaludis* strains S2, C5, C6, and C7 acquired from culture collections did not show enhanced Fe^0^ corrosion (Fig. 1(A)). The small amount of CH_4_ detected in the cultures of these strains (Fig. 1(B)) was interpreted to be synthesized by them using H_2_ generated by spontaneous Fe^0^ oxidation [see Fig. 1(C) “Control (Calc)”].

**Figure 2:**
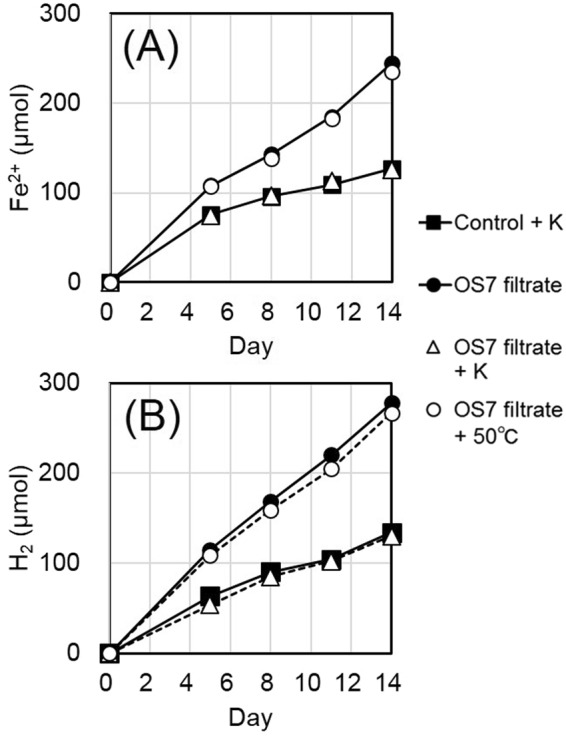
Fe^0^-corroding activities in filtrates of an OS7 culture. Filtrates of a culture of strain OS7 grown for 10 days under H_2_ + CO_2_ (80:20) were prepared, and 1 ml of the filtrate was added to 20 ml of basal medium containing Fe^0^ granules either without treatment (OS7 filtrate), or with proteinase K treatment at 50 °C for 20 min and at 100 °C for 5 min (OS7 filtrate + K), or with treatment at 50 °C for 20 min (OS7 filtrate + 50 °C). As a control, 21-ml basal medium containing Fe^0^ granules was treated with proteinase K at 50 °C for 20 min and at 100 °C for 5 min (Control + K). Duplicate samples of each treatment were incubated at 37 °C for 5, 8, 11 and 14 days, and the amounts of Fe^2+^ in basal medium **(A)**, and amounts of H_2_ in headspace gas **(B)** were determined. The amounts of CH_4_ in headspace gas were also determined, and were zero in all samples.

Previously, rapid Fe^0^ corrosion concomitant with rapid H_2_ evolution was observed with spent culture filtrates of *M. maripaludis* MM901^13^. In agreement with this, culture filtrates of strain OS7 also accelerated Fe^0^ dissolution and H_2_ evolution when they were added in basal medium containing Fe^0^ granules under a N_2_ + CO_2_ atmosphere (Fig. 2, OS7 filtrate). However, the evolution of CH_4_ was not detected in this instance. The culture filtrates treated with proteinase K at 50 °C for 20 min followed by heat-treatment at 100 °C for 5 min completely lacked the ability to enhance Fe^0^-dissolution and H_2_ evolution (OS7 filtrate + K), while the treatment of culture filtrates at 50 °C for 20 min did not affect these activities (OS7 filtrate + 50 °C). These observations suggested that the enhanced Fe^0^-dissolution (MIC activity) and H_2_ evolution were catalyzed by an enzyme such as a hydrogenase secreted from cells of strain OS7.

We noticed that strains OS7 and KA1 lost their MIC activity when they had been continuously grown under H_2_ + CO_2_. From one of such cultures of strain OS7, one clone named strain OS7mut1 was isolated by the agar shake tube technique^14,15^. As shown in Fig. 1, strain OS7mut1 exhibited neither enhanced Fe^0^ dissolution nor enhanced CH_4_ production in basal medium containing Fe^0^ granules. Furthermore, culture filtrates of strain OS7mut1 showed neither enhanced Fe^0^ dissolution nor enhanced H_2_ evolution.

### Whole genome sequencing of Fe^0^-corroding *M. maripaludis* strains

The whole genome sequences of strains OS7 and KA1 were determined. The genome of strain KA1 contained a circular 1,846,330 bp chromosome and a circular 12,084 bp plasmid, while that of strain OS7 contained a circular 1,749,749 bp chromosome but no plasmid. The G+C content of both OS7 and KA1 were 32.9 (mol)%.

We assumed that the genes involved in Fe^0^ corrosion exist in the genomes of Fe^0^-corroding strains OS7 and KA1, but are absent in the genomes of Fe^0^-non-corroding strains such as strains S2, C5, C6, and C7. We then identified genes unique to strains OS7 and KA1 by analyzing reciprocal best hits between the genomes of these strains. A total of 72 genes were identified to be specific to strains OS7 and KA1 (Table S1).

### Proteomics analyses of culture filtrates of strains OS7 and OS7mut1

Although the reciprocal best-hit approach identified 72 candidate genes involved in Fe^0^ corrosion, it was necessary to further narrow this number down. We then performed proteomic analysis of culture filtrates of strains OS7 and OS7mut1, as MIC activity was detected in the culture filtrates of strain OS7 (Fig. 2), but not in those of strain OS7mut1. Proteins in culture filtrates of strains OS7 and OS7mut1 were subjected to trypsinolysis, and analyzed by nano-ESI–MS/MS followed by Mascot searches against the gene products of the OS7 genes.

A total of 922 proteins were identified in the culture filtrate of strain OS7, many of them being cytoplasmic polypeptides, e.g. ribosomal proteins and histones. Thus, the proteomics method used was highly sensitive, and detected not only proteins actively exported into culture fluid, but also those released from lysed cells. Among the detected proteins, 59 were found only in the culture filtrate of strain OS7, but not in that of strain OS7mut1 (Table S2). An accurate estimation of protein abundance in a sample is generally difficult with MS analyses; however, a semi-quantitative method based on the protein abundance index (emPAI) is available^16^. In this method, the emPAI value is calculated by the equation: 10^PAI^-1, where PAI is the number of detected peptides divided by the theoretical number of peptides for each protein. Among these 59 proteins, the emPAI values were very low (emPAI < 1) for 56, and the 56 proteins might well be undetected in the filtrate of the OS7mut1 culture due to their low abundance. Thus, only three proteins were identified as being synthesized specifically in strain OS7 but not in strain OS7mut1. These three proteins were annotated as [NiFe] hydrogenase large and small subunits (MMOS7_11590 and MMOS7_11600, respectively), and carbonic anhydrase (MMOS7_11640) (Table S2). These three were the products of the OS7/KA1-specific genes (Table S1).

### Comparative genomics of strains OS7 and OS7mut1

**Figure 3:**
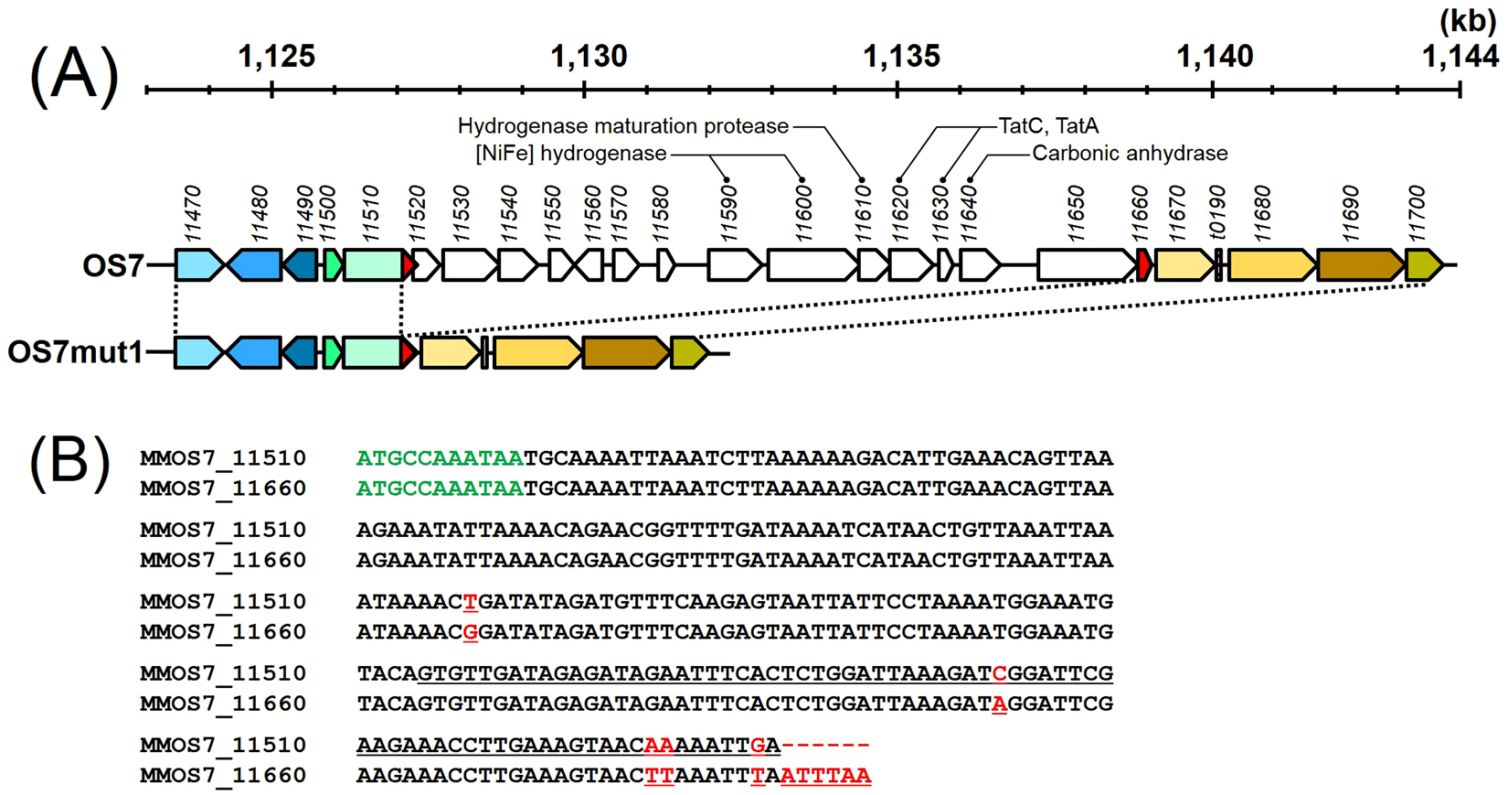
**(A)** Genome map of strain OS7 at coordinates 1,123 to 1,144 kb and that of strain OS7mut1 at the corresponding region. Numbers below the genome coordinates indicate locus tags (*MMOS7_number*). Homologous genes between the two genomes are shown in the same colors. The 3′-end sequence of *MMOS7_11510* and the 5′-end sequence of *MMOS7_11660* were almost identical; these sequences are shown in red. The gene cluster named “the MIC island” (*MMOS7_11520 - MMOS7_11650*) is absent in the genome of strain OS7mut1. **(B)** Alignment of the nucleotide sequence of the 3′-end of *MMOS7_11510* and that of *MMOS7_11660*. The upper sequence shows the 3′-end sequence of *MMOS7_11510*, the last three bases (TGA) being a stop codon, while the lower sequence shows the *MMOS7_11660* sequence, the first three bases (ATG) and the last three bases (TAA) being the initiation and stop codons, respectively. Mismatched sequences are shown in red and underlined. The 3′-end region of the nucleotide sequence of the *MMOS7_11510*/*MMOS7_11660* hybrid gene in strain OS7mut1 was 100% identical to that of *MMOS7_11660*. The nucleotide sequence of the whole *MMOS7_11510*/*MMOS7_11660* hybrid gene in strain OS7mut1 was 100% identical to the sequences of the corresponding genes in *M. maripaludis* strains, S2, C5, C6, and C7.

We also carried out Illumina sequencing of the genomes of strains OS7 and OS7mut1 to identify mutations in strain OS7mut1 responsible for the Fe^0^-non-corroding phenotype. The genome size of strain OS7mut1 was 1,737,727 bp, which was 12,022 bp shorter than that of strain OS7. When the genome sequences of strains OS7 and OS7mut1 were aligned to the reference sequence of the OS7 genome as determined by classic Sanger sequencing, a 12,022-bp region spanning *MMOS7_11520* and *MMOS7_11650* was found to be deleted in the genome of strain OS7mut1 (Fig. 3(A)). As discussed below, this DNA segment seems to be a genomic island that can spread horizontally among methanogens. Because of this reason, we call this region the “MIC island”. In addition to MIC island deletion, the Illumina sequencing detected ambiguous bases at eight different sites in the genome of strain OS7mut1; at these sites, mixed bases were identified in different reads with an abundance greater than 10% for two or more bases. Since both the wild type and mutant sequences were mixed at these eight sites, we did not treat these sites as OS7mut1-specific mutations.

At both ends of the MIC island in the genomes of strain OS7 and KA1, nearly perfect 221 bp direct repeats were found; the 3′-end sequence of *MMOS7_11510*, which was almost identical (219/221) to the *MMOS7_11660* sequence (Fig. 3(B)). This observation implied that the deletion in strain OS7mut1 had occurred by homologous recombination between the directly repeated sequences within *MMOS7_11510* and *MMOS7_11660* (Fig. S1), and that the deleted region in OS7mut1 encoded function(s) necessary for MIC activity. This notion was further supported by PCR assays for the presence/absence of the MIC-island genes, *MMOS7_11550*, *MMOS7_11570*, *MMOS7_11590*, *MOS7_11600*, and *MMOS7_116*3*0*. These genes were detected in corrosion-positive *M. maripaludis* strains KA1, OS7 and Mic1c10^4^, but not in corrosion-negative *M. maripaludis* strains, OS7mut1, S2, C5, C6, and C7.

### [NiFe] hydrogenase as a causal agent of MIC

In the deleted region, 14 putative genes were identified, of which eight genes were functionally annotated. *MMOS7_11590* and *MMOS7_11600* were annotated to encode the small and large subunits, respectively, of a [NiFe] hydrogenase. Since hydrogenases catalyze the reversible oxidation of molecular hydrogen (2 H^+^ + A_red_ ⇄ H_2_ + A_ox_), we assume that this [NiFe] hydrogenase is capable of drawing electrons from Fe^0^, generating H_2_: 2 H^+^ + Fe^0^ ⇄ H_2_ + Fe^2+^; therefore, we hypothesized that this [NiFe] hydrogenase is the causal agent of MIC. We hereafter call this [NiFe] hydrogenase the “MIC hydrogenase”.

[NiFe] hydrogenases generally consist of large and small subunits^17^. Some [NiFe] hydrogenases contain a twin-arginine translocation (Tat) signal sequence (RRxFxK) on their small subunits, and are secreted through the cytoplasmic membrane by virtue of a Tat pathway^18,19^. A Tat signal motif was found in the N-terminal region of the small subunit of the MIC hydrogenase indicating that the MIC hydrogenase was secreted across the cytoplasmic membrane. In archaea, the Tat pathway consists of TatA and TatC^18^. Interestingly, *MMOS7_11620* and *MMOS7_11630* were annotated to encode TatC and TatA, respectively. Thus, the MMOS7_11620 and MMOS7_11630 proteins were considered to form a Tat complex that recognizes the Tat secretion signal of the small subunit of the MIC hydrogenase, and the folded MIC hydrogenase (comprising the small and large subunit complexes of the MIC hydrogenase) was expected to be exported across the cytoplasmic membrane. Since the small and large subunits of the MIC hydrogenase were both detected in the culture filtrate of strain OS7 (Table S2), it is possible that the MIC hydrogenase exported across the cytoplasmic membrane is further excreted across the S-layer to the exterior of the cell.

To address this possibility, we carried out proteomics analysis of cells of strain OS7, and the emPAI values of cellular proteins per ml culture were compared with those of supernatant proteins per ml culture. Since the detection of proteins at emPAI levels below one was often not reproducible between two experiments (Table S2), 226 supernatant proteins with emPAI values higher than one were selected for the comparison. In Table S3, the emPAI values of cellular proteins collected from a 5.5-ml culture, emPAI(cell), and those of supernatant proteins collected from a 800-ml culture, emPAI(sup), are shown. These values were divided by the respective culture volumes, and the ratio, [emPAI(cells)/5.5]/[emPAI(sup)/800], was calculated for each of the 226 proteins. This ratio, relative protein abundance (cell/sup), would be less than one (with a certain degree of error) if more than half the amount of the protein is released into supernatant. The top 20 proteins showing the lowest ratio (in other words, the top 20 candidates for secreted proteins) were further examined (Table S3). The small and large subunits of the MIC hydrogenase (MMOS7_11590 and MMOS7_11600) and carbonic anhydrase (MMOS7_11640) were included in the list. Among the listed proteins, the N-terminal signal sequence was predicted in 15 proteins. Among five proteins with no signal sequence, three were small in sizes: 61 aa for MMOS7_12890 (30S ribosomal protein S27e), and 54 aa for MMOS7_15050 and MMOS7 _15070. The amino acid sequences of MMOS7_15050 and MMOS7_15070 were 100% identical, but their biological functions are unknown. These proteins were detected in the supernatant sample but not in the cell sample probably because those in the cell sample run off the bottom of a gel of SDS PAGE before they were subjected to MS/MS analysis. One of the two remaining proteins with no signal peptide was archaeal histone (MMOS7_15990) which is abundant in cells. Since the emPAI value for abundant proteins saturates^16^, it would be possible that the “relative protein abundance” value for archaeal histone shown in Table S3 was underestimated. The rest was the large subunit of [NiFe]-hydrogenase which is known to be exported by the Tat translocase as a heterodimer with the small subunit^20^. Thus, the comparison between cytoplasmic proteome and secretome allowed the identification of secreted proteins with a high degree of confidence.

### Genes required for the assembly of the MIC hydrogenase

The large subunit of [NiFe]-hydrogenases contains a NiFe(CN)_2_CO metallo-center required for the activation of H_2_, while the small subunit harbors three iron–sulfur clusters, namely a proximal [4Fe-4S] cluster, a mesial [3Fe-4S] cluster, and a distal [4Fe-4S] cluster^21,22^. For the formation of NiFe(CN)_2_CO metallo-center, six auxiliary Hyp (hydrogenase pleiotropic) proteins, namely, HypA, B, C, D, E and F, are necessary in addition to a chaperone protein, SlyD^23^. The analogs of *hypA* (*MMKA1_03080*, *MMOS7_03350*), *hypB* (*MMKA1_17480*, *MMOS7_16300*), *hypC* (*MMKA1_15530*, *MMOS7_14340*), *hypD* (*MMKA1_02960*, *MMOS7_03230*), *hypE* (*MMKA1_02810*, *MMOS7_03080)*, *hypF* (*MMKA1_01450*, *MMOS7_01710*) and *slyD* (*MMKA1_13650* or *MMKA1_06200*, *MMOS7_12980* or *MMOS7_06150*), were all found in the chromosomes of strains OS7 and KA1. After the insertion of the metallo-center, an endopeptidase removes the C-terminal extension of the large subunit. A gene for such an endopeptidase (*MMOS7_11610*) was found next to the structural genes for the MIC hydrogenase. In *Escherichia coli* expressing at least three [NiFe] hydrogenases, a single set of HypA-HypF proteins are involved in the insertion of the metallo-center in the three hydrogenases. However, for the cleavage of their C-terminal extension, each hydrogenase requires its own specific endopeptidase encoded within the same operon^17^. This rule could also be applied to the MIC hydrogenase.

### MIC hydrogenase is a novel family member of [NiFe] hydrogenases

To date, [NiFe] hydrogenases have been classified into four groups based on their amino acid sequences, the organization of their genes, and their biochemical characteristics^17,24^. Phylogenetic analysis of the large and small subunits of the MIC hydrogenase revealed that these proteins were located outside the four known groups of [NiFe] hydrogenases indicating that the MIC hydrogenase is a novel type of [NiFe] hydrogenase (Fig. S2).

The alignment of the small subunit of the MIC hydrogenase with published small subunit sequences revealed that the residues coordinating the proximal [4Fe-4S], mesial [3Fe-4S], or distal [4Fe-4S] cluster were well conserved in the small subunit of the MIC hydrogenase (Fig. S3). In the active form of the NiFe(CN)_2_CO metallo-center in the large subunit of [NiFe] hydrogenases, the nickel atom is coordinated either with two cysteines or with one selenocysteine and one cysteine, while the iron atom is bridged to the nickel atom by two cysteinyl sulfur ligands^20,21^. These amino acids were conserved, while no selenocysteine was found in the large subunit of the MIC hydrogenase (Fig. S4).

### Model of hydrogenase-induced Fe^0^ corrosion

**Figure 4:**
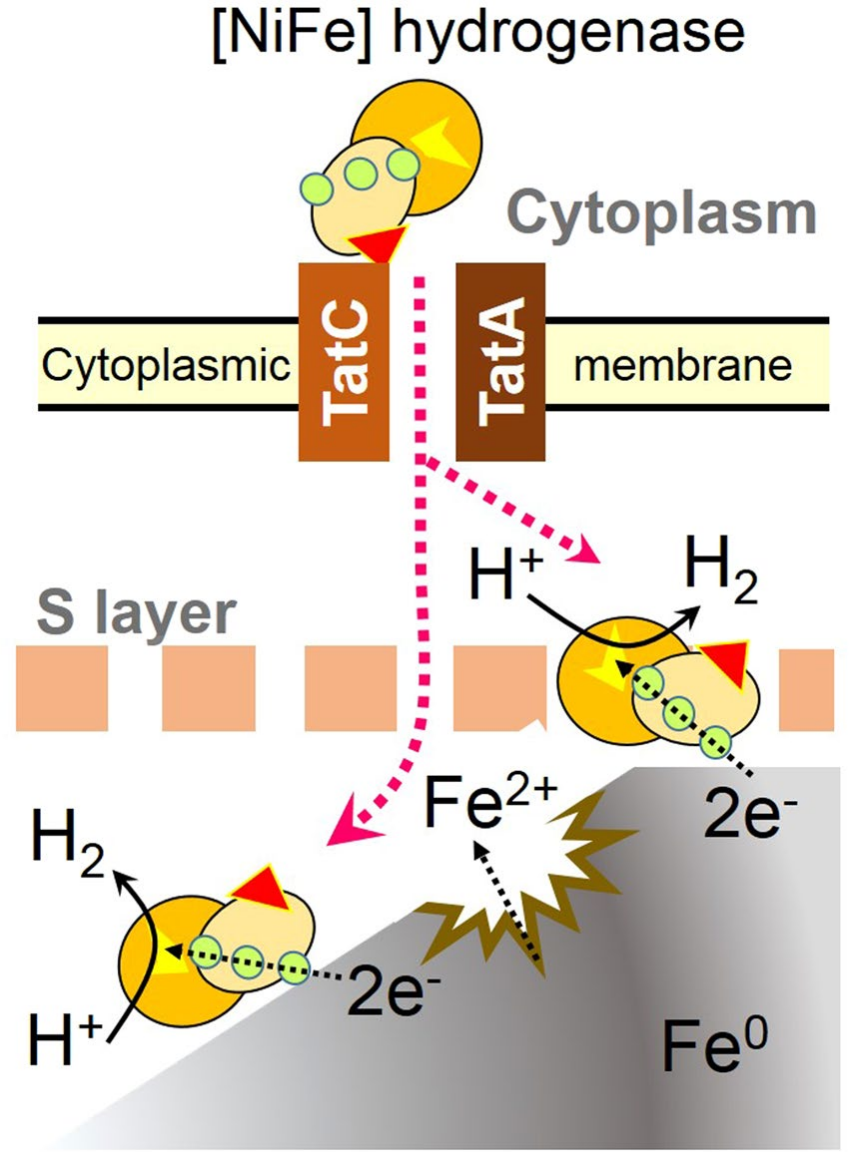
Model of Fe^0^ corrosion enhanced by MIC hydrogenase. Two spheres of a [NiFe] hydrogenase represent the large and small subunits. A yellow patch on the large subunit represents the catalytic center that catalyzes reversible oxidation of H_2_. Three circles on the small subunit represent proximal, mesial, and distal iron-sulfur clusters that transfer electrons from an electron donor to the catalytic center to form H_2_. A red triangle on the small subunit represents a Tat signal peptide that is recognized by TatC. TatA and TatC constitute the TatA/TatC translocase through which the MIC hydrogenase is secreted through the cytoplasmic membrane. After completion of the translocation, the signal peptide is removed by a signal peptidase. The MIC hydrogenase localizes either on the surface of, or outside, the S-layer to receive electrons from Fe^0^.

Our model of the MIC-hydrogenase-induced Fe^0^ corrosion, which is similar to those proposed previously^5,13^ is depicted in Fig. 4. We postulate that the MIC hydrogenase directly contacts the Fe^0^ surface, where the hydrogenase receives electrons from Fe^0^ to form H_2_ using H^+^ from water. To be able to do so, the MIC hydrogenase should either be excreted outside the cell or be presented at the external surface of the S-layer. As already discussed above, the MIC hydrogenase was predicted to be secreted in a Tat-dependent manner. Furthermore, we showed that the enzyme was excreted in culture medium (Fig. 2 and Table S3). However, it is still unclear whether the excretion of the MIC hydrogenase occurs either through the intact S layer or through damaged S-layer structure.

For proton reduction by [NiFe] hydrogenases, electrons are transferred from a redox partner, such as NADH, reduced ferredoxin or reduced cytochrome *c*_*3*_, to the Ni-Fe active site via three (distal, mesial and proximal) iron-sulfur clusters^21,22^. The distal iron-sulfur cluster is exposed to the molecular surface, and can directly interact with a specific redox partner. Thus, it is interesting to resolve the 3D structure of the MIC hydrogenase to understand how this enzyme contacts the Fe^0^ surface. In addition, the determination of redox potentials of the three iron-sulfur clusters of the MIC hydrogenase would be important, as these iron-sulfur clusters should have appropriate redox potentials for the efficient delivery of electrons from Fe^0^ to H^+^ via the catalytic center of the MIC hydrogenase^25^.

Several studies have reported that hydrogenases from different microorganisms stimulated the oxidation of Fe^0^ with concomitant hydrogen evolution^5,26^. In addition, other redox enzymes such as formate dehydrogenase^13^ and heterodisulfide reductase supercomplex^27^ also stimulate the Fe^0^ oxidation. From these observations, certain redox enzymes seemed to be able to oxidize Fe^0^ when they come into contact with the Fe^0^ surface. However, redox enzymes which do not possess Tat or Sec-dependent signal sequences cannot interact with external Fe^0^. Thus, one of the reasons why most hydrogenase-bearing microorganisms are not Fe^0^-corrosive in laboratory conditions may be because they do not express extracellular redox enzymes. It should however be noted that microorganisms that do not secrete extracellular redox enzymes could also be involved in MIC in natural environments, e.g. in biofilms developed on metal substrata, through release of redox enzymes from lysed cells^13^.

### Possible roles of γ-carbonic anhydrase in MIC

A gene encoding γ-carbonic anhydrase (MMOS7_11640) bearing a *sec*-dependent signal peptide at its N-terminal region was identified within the MIC island, suggesting that this enzyme was secreted across the cytoplasmic membrane. This inference was confirmed by proteomics analyses (Tables S2 and S3). Carbonic anhydrases catalyze the reversible hydration of CO_2_ to bicarbonate: CO_2_ + H_2_O ⇄ HCO_3_^−^ + H^+^. Since CO_2_ is an indispensable electron acceptor in methanogens, carbonic anhydrase in the cytoplasm may be important to convert bicarbonate to CO_2_, which is then used in the first committed step of methanogenesis. On the other hand, the role of extracellular carbonic anhydrases is not clear, one possibility being the conversion of CO_2_ to bicarbonate, which can be transported within cells by a bicarbonate transporter^28^.

In the presence of Fe^2+^ and bicarbonate, ferrous carbonate is formed: Fe^2+^ + HCO_3_^−^ = FeCO_3_ + H^+^. Under such conditions, the external carbonic anhydrase could contribute indirectly to the formation of FeCO_3_ by converting CO_2_ into bicarbonate. However, more studies are needed to elucidate the roles of the extracellular carbonic anhydrase in the Fe^0^ corrosion and the proliferation of methanogens containing the MIC island.

### Genetically unstable MIC island

We concluded that the MIC island is unstable in the genomes of strains OS7 and KA1 from the following two observations. First, in an early stage of the KA1 genome sequencing study using the Sanger method, we found two different genes downstream of the *MMOS7_11510* ortholog. In some shotgun clones, the downstream gene was an *MMOS_11520* ortholog, as seen in the genome of strain OS7, while the *MMOS_11670* ortholog was the downstream gene in other shotgun clones as seen in the genome of strain OS7mut1 (Fig. 3). We interpreted this observation as indicating that a culture of strain KA1 from which DNA was prepared for the genome sequencing study was heterogeneous, and contained wild-type cells and spontaneous deletion mutants lacking the MIC-island. Second, cultures of strain OS7 grown under H_2_ + CO_2_ often showed very low MIC activities suggesting that a significant proportion of the cell population in culture lost the MIC island. In fact, strain OS7mut1 was isolated from one of such cultures.

We thus inferred that the MIC island could readily be excised through homologous recombination at the direct repeat sequences, excising a circular DNA and leaving a hybrid gene of *MMOS7_11510* and *MMOS7_11660* in the chromosome (Fig. S1(A)). The function of *MMOS7_11510* is not clear. It encodes a protein belonging to the family of “putative methanogenesis marker protein 1” according to the INTERPRO classification (IPR017667), which is widely distributed in methanogens.

Once the excised circular DNA is transferred into other hosts through conjugation, transduction, or transformation, the MIC island can be integrated into the chromosome of the new host at the *MMOS7_11510*-ortholog site via homologous recombination (Fig. S1(B)). Thus, the genetic instability of the MIC island provides a means of horizontal transfer of the MIC island among methanogens.

As described in the Introduction section, the Fe^0^-corroding activities in two strains of *M. maripaludis*, MM901 and MM1284, were investigated by Deutzmann *et al*.^13^. Strain MM901 was isolated from strain S2 by disrupting the uracil phosphoribosyltransferase gene, while strain MM1284 was isolated from strain MM901 by disrupting five hydrogenase genes (*vhuU*, *vhcA*, *fruA*, *frcA*, *hmd*, and *ehbN*) and one dehydrogenase gene (*hmd*). Strain MM901 showed enhanced Fe^0^-corroding activity, whereas strain MM1284 did not. These results are inconsistent to ours. As shown in Fig. 1, strain S2, which is the parental strain of MM901, did not exhibit Fe^0^-corroding activity. Strain S2 carries genes for the subunits of six [NiFe] hydrogenases, namely the subunit genes for two coenzyme F_420_-reducing hydrogenases (*Mmp1382 – 1385*; and *Mmp0817 - 0820*), those for two non-F_420_-reducing hydrogenases (*vhu*: *Mmp1692 – 1696*; and *vhc*: *Mmp0821 - 0824*) and those for two energy-conserving hydrogenases (*eha*: *Mmp1448 -1467*; and *ehb*: *Mmp0400*, 0940, 1049, 1073, 1074, 1153, 1469, and *1621–1629*). This strain also carries two H_2_-dependent methylene tetrahydromethanopterin dehydrogenase genes (*hmd*: *Mmp0127*; and *hmd* paralog: *Mmp1716*)^29^. Strains KA1, OS7, and OS7mut1 carry orthologs of all the genes. Nevertheless, strain OS7mut1 was deficient in Fe^0^-corroding activity.

We attributed these contradictory results to the instability of the MIC island. We hypothesize that strain S2 originally possessed the MIC island, as did strain MM901, but the MIC island in strain S2 was lost before we acquired it. Similarly, the MIC island in strain MM1284 was lost before Deutzmann *et al*.^13^ performed their experiments. Strain S2 has been usually maintained and distributed with H_2_ as a sole electron donor. This cultivation condition does not prevent the loss of Fe^0^-corrosion ability as described before. To avoid the accumulation of MIC-island-free derivatives, we often purify colonies of strains OS7 and KA1 by the agar shake tube technique, and MIC-positive clones were preserved in basal medium supplemented with 20% glycerol at −80 °C. This precaution is very important for researchers studying MIC-inducing methanogens, and possibly MIC-inducing SRBs.

### Evolution of the MIC island

**Figure 5:**
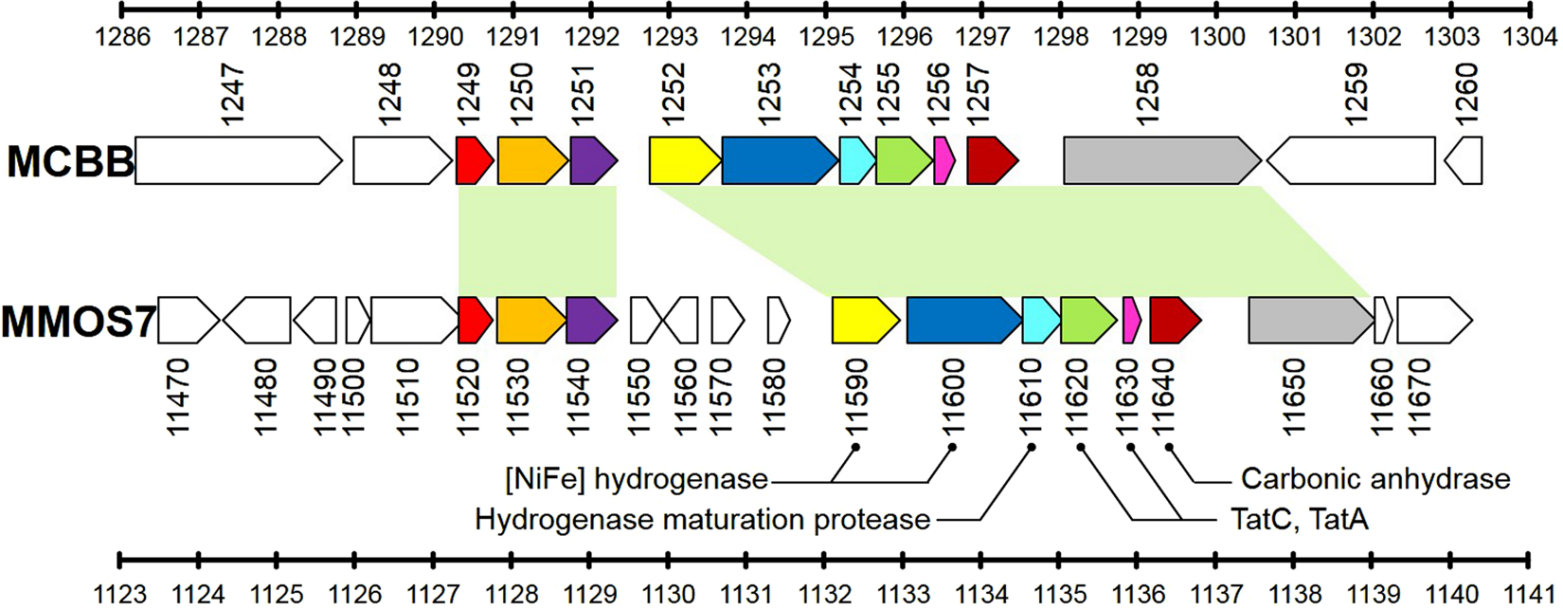
Genome map of strain Buetzberg at coordinates 1,286 to 1,304 kb and that of strain OS7 at 1,123 to 1,141 kb. The upper part shows genes of strain Buetzberg (*MCBB_1247* to *MCBB_1260*), while the lower part shows genes of strain OS7. Both regions contained homologous genes that are shown in the same colors.

Recently, the genome sequence of *Methanobacterium congolense* strain Buetzberg was published^30^. Homology at the nucleotide sequence level was not high between the genome sequence of strain OS7 and that of strain Buetzberg, with the exceptions of several limited regions. One such region showed extensive homology to the MIC island (Fig. 5) indicating that gene clusters related to the MIC island have spread among methanogens beyond the genus level.

Careful comparison of the nucleotide sequences of the MIC islands in strains OS7/KA1 and strain Buetzberg gave an insight into how the MIC island in strain OS7/KA1 has evolved. As shown in Fig. 5, the first gene of the MIC island, *MMOS7_11520*, was homologous to *MCBB_1249*, while the last gene of the MIC island, *MMOS7_11650*, was homologous to the 5′-half of *MCBB_1258* (Figs 5 and S5). *MCBB_1258* encoded two types of domains, PAS and GAF; both are found in many proteins involved in signal transduction, while *MMOS7_11650* only encoded the PAS domain. Thus, we considered *MCBB_1258* as representing the conserved ancestral gene structure, while *MMOS7_11650* is a truncated derivative of the ancestral gene. Four genes, *MMOS7_11550*, *MMOS7_11560*, *MMOS7_11570*, and *MMOS7_11580*, found in the MIC island of strain OS7, were absent in the island of strain Buetzberg. These four genes might have been either acquired in the island of strain OS7 by horizontal gene transfer, or deleted from an ancestral island in strain Buetzberg.

The 74 bp-long 3′-end sequence of *MMOS7_11510* overlaps with the 5′-end of *MMOS7_11520*, which is the first gene of the MIC island in strain OS7 [Fig. S5(A), underlined sequence]. As mentioned previously, the 3′-end sequence of *MMOS7_11510* was almost identical to the *MMOS7_11660* sequence except for the last nine nucleotides: [AAAAATTGA; Fig. 3(B)]. This AAAAATTGA sequence was rather similar to the aligned *MCBB_1249* sequence [Figure S5(A), green underlined sequence]. This observation suggested that the 3′-end sequence of an ancestral *MMOS7_11510* locus was TTAAATTTAATTTAA found in *MMOS7_11660* [Figs 3(B) and S5(B)], and that the 5′-end of the current MIC island in strain OS7 (the *MMOS7_11520*) had been formed by the fusion between the ancestral *MMOS7_11510* with the deletion at the 3′-end (TTAAATTTAATTTAA) and an ancestral *MCBB_1249* (the first gene of an ancestral MIC island) with a deletion of more than 100 bp at the 5′-end (Fig. S5(B)).

The 3′-end sequence of *MMOS7_11650* (ATGCCAAATAA: bold and underlined sequence in Fig. S6) which corresponds to the 3′-end region of the MIC island in strain OS7 overlaps with the 5′-end of *MMOS7_11660*. Since the ATGCCAAATAA sequence was also found in *MMOS7_11510* [Fig. 3(B), green sequence], we inferred that the ATGCCAAATAA sequence existed in the ancestral *MMOS7_11510*. On the other hand, *MCBB_1258* has the ATGCCAAA(GAT) sequence at the corresponding site (Fig. S6, red sequence). Hence, the 3′-end of the current MIC island in strain OS7 (the *MMOS7_11650-11660* junction) would be formed by the fusion of the ancestral *MCBB_1258* and the ancestral *MMOS7_11510* by homologous recombination at the ATGCAAA sequence. These inferences are summarized in Fig. S7.

In the MIC island, the genes for the small and large subunits of the MIC hydrogenase, a TatA/TatC translocase, and a hydrogenase maturation protease are encoded. The clustering of the genes required for the functional expression of the MIC hydrogenase in a single genomic island may provide evolutionary advantages as the maturation of a [NiFe] hydrogenase requires a specific hydrogenase maturation protease^31^, and the Tat export system is not common in methanogens^19^. The presence of the MIC hydrogenase thus may increase the fitness of methanogens thriving in environments with access to steel structures, where Fe^0^ is available as a source of electrons.

Horizontal gene transfer plays an important role in the evolution of microorganisms, where genomic islands encoding various functions contribute to rapid propagation of specific traits among prokaryotes in local environments^32^. As discussed above, the MIC island can be transferred into other hosts through a homologous recombination-mediated mechanism (Fig. S1). However, the MIC island seems to encode no enzyme involved in transposition. Therefore, horizontal transfer events of the MIC island may occur at a very low frequency. On the other hand, methanogens possessing the MIC island, once acquired, would be enriched in environments where Fe^0^ is available as a source of electron, and increase the risk of MIC with time. Since the increasing occurrence of methanogens possessing the MIC island is a global industrial concern, the presence, persistence, and potential transfer of the MIC island among populations should be investigated in model environments such as pipelines, in parallel to the development of methods to control MIC-causing methanogens. Our findings provide a basis for the development of molecular tools to monitor epidemiology, and to perform surveillance and risk assessment of MIC-inducing *M. maripaludis*.

## Materials and Methods

### Strains, media, and culture conditions

*M. maripaludis* strains, KA1^11^ (=NBRC 102054), S2^29,33^ (=NBRC 101832), C5^33^ (=NBRC 102114), C6^33^ (=JCM 10012), and C7^33^ (=JCM 10012) were obtained from NITE Biological Resource Center (https://www.nite.go.jp/en/nbrc/index.html) and Japan Collection of Microorganisms (http://jcm.brc.riken.jp/en/), while *M. maripaludis* strains OS7 (=NBRC 103642) and OS7mut1 (=NBRC 105638) were isolated in this study.

Basal medium, which is “Artificial seawater medium”^34^ supplemented with 100 mM sodium 2-[4-(2-Hydroxyethyl)-1-piperazinyl] ethanesulfonate (pH 7.0) and 0.1% (w/v) L-cysteine hydrochloride monohydrate, was used to cultivate *M. maripaludis* strains with two different methods. In the first method, where H_2_ was an electron donor and CO_2_ was an electron acceptor, cells were cultivated at 37 °C in a 70 ml serum bottle containing 20 ml basal medium under a H_2_ + CO_2_ gas mixture (80:20, 1.78 atm). In the second method, where Fe^0^ was an electron donor and CO_2_ was an electron acceptor, cells were cultivated at 37 °C in a 70 ml serum bottle containing 20 ml basal medium and 6 g of Fe^0^ granules (1 to 2 mm in diameter and 99.98% purity) under the N_2_ + CO_2_ gas mixture (80:20, 1 atm). The bottles were sealed with butyl rubber stoppers.

### Determination of Fe^0^-corroding activities of *M. maripaludis* strains and their culture filtrates

One milliliter of a preculture of strain OS7, OS7mut1, S2, C5, C6, or C7 grown in 20 ml of basal medium under H_2_ + CO_2_ at 37 °C for 10 days was transferred into 20 ml basal medium containing Fe^0^ granules, and cultivated under N_2_ + CO_2_ at 37 °C for 14 days. A sterile control was also incubated under the same conditions. The concentrations of Fe^2+^, CH_4_, and H_2_ generated from the cultures were determined as described previously^11^. All assays were conducted using four replicates.

A culture of strain OS7 grown at 37 °C for 10 days in 20 ml basal medium under H_2_ + CO_2_ was filtered through a 0.22 µm filter, and 1 ml of the filtrate was added to 20 ml basal medium containing Fe^0^ granules under N_2_ + CO_2_. The medium containing the filtrate was either untreated (OS7 filtrate), or treated either with 100 μl of proteinase K (Qiagen; >600 mAU/ml; one mAU releases 1 µmol tyrosine per min from hemoglobin at 37 °C, pH7.5) at 50 °C for 20 min followed by the heat treatment at 100 °C for 5 min (OS7 filtrate + K), or without Qiagen proteinase K at 50 °C for 20 min (OS7 filtrate +50 °C). As a sterile control, 21 ml basal medium containing Fe^0^ granules was treated with 100 μl Qiagen proteinase K under N_2_ + CO_2_ at 50 °C for 20 min followed by the treatment at 100 °C for 5 min (Control + K). The duplicated samples thus prepared were incubated at 37 °C for 14 days under N_2_ + CO_2_, and the concentrations of Fe^2+^, CH_4_, and H_2_ in the samples were determined as described above.

### Omics studies

The methods employed for genome and proteome analyses are described in the Supplementary Materials and Methods.

## Electronic supplementary material

Electronic supplementary materials

## Data Availability

Complete genome sequences of *M. maripaludis* strains OS7, OS7mut1, and KA1 were deposited in DDBJ/EMBL/GenBank under the accession numbers AP011528, CP020120, and AP011526, respectively. Other datasets generated during and/or analyzed during the current study are available from the corresponding author on reasonable request.
