# Supplementary material for: An extracellular [NiFe] hydrogenase mediating iron corrosion is encoded in a genetically unstable genomic island in Methanococcus maripaludis
Source: Sci Rep. 2018 Oct 11;8:15149. doi: 10.1038/s41598-018-33541-5 (PMC6181927; doi:10.1038/s41598-018-33541-5)
Supplement: Supplementary file 1 — Electronic supplementary materials [file 41598_2018_33541_MOESM1_ESM.docx]

**Electronic supplementary materials**

Title: An extracellular [NiFe] hydrogenase mediating iron corrosion is encoded in a genetically unstable genomic island in *Methanococcus maripaludis*

Authors: Hirohito Tsurumaru, Naofumi Ito, Koji Mori, Satoshi Wakai, Taku Uchiyama, Takao Iino, Akira Hosoyama, Hanako Ataku, Keiko Nishijima, Miyako Mise, Ai Shimizu, Takeshi Harada, Hiroshi Horikawa, Natsuko Ichikawa, Tomohiro Sekigawa, Koji Jinno, Satoshi Tanikawa, Jun Yamazaki, Kazumi Sasaki, Syuji Yamazaki, Nobuyuki Fujita, Shigeaki Harayama

NITE Biological Resource Center (NBRC), National Institute of Technology and Evaluation (NITE), Chiba 292-0818, Japan

*Corresponding author: Shigeaki Harayama, Research and Development Initiative, 1-13-27 Kasuga, Bunkyo-ku, Tokyo 112-8551, Japan

Tel.: +81-3-3817-7360; fax: +81-3-3817-7102

E-mail address: [harayama@bio.chuo-u.ac.jp](mailto:harayama@bio.chuo-u.ac.jp)

**Supplementary materials and methods**

**Whole-genome sequencing and assembly of the KA1, OS7, and OS7mut1 genome sequences**

Strains OS7 and KA1 were precultured at 37°C for 10 days in basal medium containing Fe^0^ granules under N_2_ + CO_2_, and 10 ml of each culture was transferred into 400 ml of basal medium in a 1,380 ml butyl rubber-stoppered bottle, and cultivated under H_2_ + CO_2_ at 37°C for 10 days. Cells were collected by centrifugation at 20,000 × *g* for 10 min, and the genomic DNA of strains OS7 and KA1 were extracted by the method of Argyle *et al*.^1^. The complete genome sequences of strains OS7 and KA1 were determined by the so-called “classical whole-genome shotgun sequencing strategy”, using methods described previously^2-4^.

The complete genome sequences of strains OS7 and mutant OS7mut1 were determined using next-generation sequencing technology as follows. Strain OS7 was precultured in basal medium containing Fe^0^ granules under N_2_ + CO_2_, while the strain OSmut1, which cannot use Fe^0^ as an electron donor, was precultured in basal medium under H_2_ + CO_2_. 10 ml of each preculture was transferred into 400 ml of basal medium in a 1,380-ml butyl rubber-stoppered bottle, and the 400-ml culture was cultivated under H_2_ + CO_2_ at 37°C for 10 days. DNA of strains OS7 and OS7mut1 was extracted using the EZ1 DNA Tissue Kit (Qiagen), and fragmented using an ultrasonicator (Covaris S220) to generate DNA fragments. A library for sequencing was prepared by means of the TruSeq DNA Library Prep Kit (Illumina), and the library was paired-end sequenced (2 × 250 bp) on a MiSeq instrument (Illumina) using a MiSeq reagent kit v2 (Illumina). The MiSeq reads were trimmed using sickle software (https://github.com/najoshi/sickle), and the trimmed reads (145 Mb) were assembled *de novo* using Newbler 3.0 software (Roche Diagnostics) with default parameters. The Newbler contigs with an average coverage of 81.5-fold were assembled into a single scaffold using Sequencher 5.1 software (Gene Codes Corporation). Almost all gaps in the scaffold were filled with paired-end information provided by Sequencher. The remaining gaps that contain repetitive regions such as rRNA operons were filled by aligning them to the genome sequence of strain OS7, as determined by the Sanger method (= the reference sequence). Finally, mutation sites (insertions, deletions, or base substitutions) in strain OS7mut1 were identified by means of Sequencher.

**Functional annotation of genes in the genomes of strains OS7 and KA1**

Open reading frames (ORFs) of strains OS7 and KA1 were predicted with GeneMarkS^5^, Glimmer^6^, and Glimmer 3^7^ software. The predicted ORFs were manually annotated, primarily by means of the results of BLASTp searches^8^ against the UniProt database (http://www.uniprot.org/). Conserved domains were identified using the NCBI conserved domain search software (http://www.ncbi.nlm.nih.gov/Structure/cdd/wrpsb.cgi). Sec-dependent signal peptides were predicted using the SignalP 3.0 Server (http://www.cbs.dtu.dk/services/SignalP-3.0/)^9^, while twin-arginine (Tat) signal peptides and cleavage sites in the Tat peptides were predicted by the TatP 1.0 Server program (http://www.cbs.dtu.dk/services/TatP/)^10^. Putative untranslated genes were identified by Rfam^11^ and tRNAscan-SE^12^ programs. ORFs in the genome of strain OS7 were compared to those in the genomes of strains KA1, S2, (GenBank: BX950229), C5 (GenBank: CP000609), C6 (GenBank: CP000867), and C7 (GenBank: CP000745) using BLASTn analysis^12^ to detect orthologs as reciprocal best hits. Phylogenetic analysis of proteins was conducted by the neighbor-joining method using MEGA6 software (http://www.megasoftware.net)^13^.

**Proteomic analyses**

Strains OS7 and OS7mut1 were respectively cultivated in 800 ml basal medium in two 1,380 ml butyl rubber-stoppered bottles and grown under H_2_ + CO_2_ at 37°C for 10 days. Cells of strain OS7 were collected by centrifugation from a 5.5-ml culture, and cellular proteins were isolated as described previously^14^. The supernatant of 800-ml culture was passed through a 0.2 µm membrane filter, and proteins in the filtrate were precipitated by centrifugation after adding 0.1 volume of trichloroacetic acid. The cellular proteins and the proteins collected from the filtrates were resolubilized in 50 µl of buffer consisting of 7 M urea, 2 M thiourea, 50 mM dithiothreitol, 4 % (w/v) 3-[(3-cholamidopropyl) dimethylammonio]-1-propanesulfonate, and 40 mM tris(hydroxymethyl)aminomethane-HCl (pH 6.8), and separated by sodium dodecyl sulphate polyacrylamide gel electrophoresis (SDS-PAGE). Each of the gel for the cellular or culture-filtrate protein samples was cut into 100 or 10 slices, respectively, and proteins in these gel slices were digested with trypsin. The resulting peptides were then separated by a Paradigm MS4 HPLC system (Michrom Bioresources) fitted with a C_18_ reverse-phase column (50 mm length and 0.2 mm internal diameter) which was coupled to a LTQ ion trap mass spectrometer (Thermo Finnigan) equipped with an ADVANCE nano-electrospray ionization (nano-ESI) source (AMR, Tokyo).

MS/MS spectra were searched with the MASCOT database search software (Matrix Science) against the OS7 gene database. Peptides having a MASCOT ion score > 63 were selected as matched peptides, and proteins with two or more matched peptides were considered as valid identifications. Semi-quantitative estimation of abundances of proteins thus identified was done using "exponentially modified protein abundance index (emPAI)" as described previously^15^. The proteomics data have been deposited in the jPOSTrepo (Japan ProteOme STandard Repository) with the data set identifier JPST000448.

**Supplementary tables**

| **Table S1**  Genes specific to iron-corroding *Methanococcus maripaludis* strains OS7 and KA1 | |  |
| --- | --- | --- |
| Gene name (locus tag)  in strain OS7 ^a^ | Deduced gene product |  |
|  |  |  |
|  |  |  |
| *MMOS7_01110* | coenzyme F420 hydrogenase/dehydrogenase beta subunit |  |
| *MMOS7_01120* | putative sulfite reductase iron-sulfur binding protein |  |
| *MMOS7_01130* | coenzyme F420-dependent sulfite reductase |  |
| *MMOS7_01140* | putative transcriptional regulator TrmB |  |
| *MMOS7_02230* | hypothetical protein |  |
| *MMOS7_02250* | hypothetical protein |  |
| *MMOS7_02710* | hypothetical protein |  |
| *MMOS7_02730* | hypothetical protein |  |
| *MMOS7_02740* | hypothetical protein |  |
| *MMOS7_03530* | hypothetical protein |  |
| *MMOS7_04380* | hypothetical protein |  |
| *MMOS7_04410* | hypothetical protein |  |
| *MMOS7_04890* | hypothetical protein |  |
| *MMOS7_04900* | hypothetical protein |  |
| *MMOS7_04940* | resolvase |  |
| *MMOS7_05100* | putative addiction module toxin, RelE/StbE |  |
| *MMOS7_05110* | putative iron compound ABC transporter periplasmic binding protein |  |
| *MMOS7_05120* | putative iron compound ABC transporter ATP-binding protein |  |
| *MMOS7_05130* | hypothetical protein |  |
| *MMOS7_05150* | magnesium-chelatase subunit ChlI |  |
| *MMOS7_05160* | putative magnesium-chelatase subunit ChlH |  |
| *MMOS7_05180* | hypothetical protein |  |
| *MMOS7_05190* | hypothetical protein |  |
| *MMOS7_05200* | putative cellulosome anchoring protein |  |
| *MMOS7_05210* | putative iron compound ABC transporter periplasmic binding protein |  |
| *MMOS7_05220* | putative iron compound ABC transporter permease protein |  |
| *MMOS7_05230* | putative iron compound ABC transporter permease protein |  |
| *MMOS7_05240* | hypothetical protein |  |
| *MMOS7_05260* | hypothetical protein |  |
| *MMOS7_05270* | hypothetical protein |  |
| *MMOS7_06040* | hypothetical protein |  |
| *MMOS7_06170* | hypothetical protein |  |
| *MMOS7_06570* | hypothetical protein |  |
| *MMOS7_08040* | type I restriction-modification system S-subunit |  |
| *MMOS7_08130* | hypothetical protein |  |
| *MMOS7_08390* | putative site-specific recombinase |  |
| *MMOS7_08400* | hypothetical protein |  |
| *MMOS7_08410* | hypothetical protein |  |
| *MMOS7_08420* | hypothetical protein |  |
| *MMOS7_08460* | hypothetical protein |  |
| *MMOS7_08730* | hypothetical protein |  |
| *MMOS7_08750* | hypothetical protein |  |
| *MMOS7_08760* | hypothetical protein |  |
| *MMOS7_08790* | hypothetical protein |  |
| *MMOS7_09110* | putative type II restriction enzyme methylase |  |
| *MMOS7_09400* | hypothetical protein |  |
| *MMOS7_09790* | hypothetical protein |  |
| *MMOS7_10320* | hypothetical protein |  |
| *MMOS7_10840* | hypothetical protein |  |
| *MMOS7_10910* | hypothetical protein |  |
| *MMOS7_10920* | hypothetical protein |  |
| *MMOS7_11520* | putative GTP-binding protein |  |
| *MMOS7_11530* | hypothetical protein |  |
| *MMOS7_11540* | putative endonuclease III |  |
| *MMOS7_11550* | hypothetical protein |  |
| *MMOS7_11560* | hypothetical protein |  |
| *MMOS7_11570* | hypothetical protein |  |
| *MMOS7_11580* | hypothetical protein |  |
| *MMOS7_11590* | putative [NiFe] hydrogenase small subunit |  |
| *MMOS7_11600* | putative [NiFe] hydrogenase large subunit |  |
| *MMOS7_11610* | putative hydrogenase maturation protease |  |
| *MMOS7_11620* | sec-independent protein translocase protein TatC |  |
| *MMOS7_11630* | sec-independent protein translocase protein TatA |  |
| *MMOS7_11640* | carbonic anhydrase |  |
| *MMOS7_11650* | putative sensor protein |  |
| *MMOS7_11910* | glycosyl transferase |  |
| *MMOS7_14830* | hypothetical protein |  |
| *MMOS7_14950* | hypothetical protein |  |
| *MMOS7_16240* | hypothetical protein |  |
| *MMOS7_16370* | hypothetical protein |  |
| *MMOS7_16880* | hypothetical protein |  |
| *MMOS7_18080* | coenzyme F420-non-reducing hydrogenase subunit U |  |
| ^a^ Gray indicates genes in the MIC island. The MIC island was deleted in strain OS7mut1. | | |

**Table S2**

Proteins detected in culture filtrates of strain OS7 but not in those of strain OS7mut1

| **Locus tag** | **emPAI** |
| --- | --- |
| MMOS7_00160 | 0.15 |
| MMOS7_00510 | 0.02 |
| MMOS7_00640 | 0.12 |
| MMOS7_00650 | 0.22 |
| MMOS7_00900 | 0.26 |
| MMOS7_01270 | 0.15 |
| MMOS7_01930 | 0.17 |
| MMOS7_02050 | 0.06 |
| MMOS7_02180 | 0.11 |
| MMOS7_02380 | 0.26 |
| MMOS7_03100 | 0.07 |
| MMOS7_03140 | 0.23 |
| MMOS7_03380 | 0.09 |
| MMOS7_03630 | 0.13 |
| MMOS7_04540 | 0.09 |
| MMOS7_04560 | 0.21 |
| MMOS7_06310 | 0.12 |
| MMOS7_06750 | 0.06 |
| MMOS7_06840 | 0.10 |
| MMOS7_07520 | 0.07 |
| MMOS7_08030 | 0.02 |
| MMOS7_08520 | 0.08 |
| MMOS7_08810 | 0.19 |
| MMOS7_08830 | 0.06 |
| MMOS7_08930 | 0.15 |
| MMOS7_10380 | 0.10 |
| MMOS7_11160 | 0.10 |
| MMOS7_11590 | 6.63 |
| MMOS7_11600 | 4.95 |
| MMOS7_11640 | 15.38 |
| **Locus tag** | **emPAI** |
| MMOS7_12360 | 0.04 |
| MMOS7_12400 | 0.15 |
| MMOS7_12410 | 0.13 |
| MMOS7_12900 | 0.21 |
| MMOS7_12910 | 0.30 |
| MMOS7_13280 | 0.06 |
| MMOS7_13350 | 0.12 |
| MMOS7_13470 | 0.19 |
| MMOS7_14100 | 0.12 |
| MMOS7_14340 | 0.47 |
| MMOS7_14380 | 0.14 |
| MMOS7_14410 | 0.26 |
| MMOS7_14580 | 0.52 |
| MMOS7_15010 | 0.15 |
| MMOS7_15910 | 0.15 |
| MMOS7_16160 | 0.41 |
| MMOS7_16190 | 0.15 |
| MMOS7_16330 | 0.14 |
| MMOS7_16430 | 0.15 |
| MMOS7_16490 | 0.39 |
| MMOS7_16500 | 0.12 |
| MMOS7_16660 | 0.08 |
| MMOS7_16850 | 0.21 |
| MMOS7_17260 | 0.47 |
| MMOS7_17340 | 0.14 |
| MMOS7_17360 | 0.18 |
| MMOS7_17430 | 0.04 |
| MMOS7_17730 | 0.29 |
| MMOS7_17990 | 0.78 |
| MMOS7_18040 | 0.19 |

emPAI is an index for protein abundance. Among the proteins detected in culture filtrates of strain OS7 but not in those of strain OS7mut1, only three proteins (shaded), MMOS7_11590, MMOS7_11600, and MMOS7_11640 encoding [NiFe] hydrogenase small subunit, [NiFe] hydrogenase large subunit, and carbonic anhydrase, respectively, were found in abundance.

**Table S3**

Identification of secreted proteins by comparing intracellular and extracellular protein abundance

|  | ORF | Signal sequence^*^ | emPAI | | Relative protein abundance (cell/sup)^****^ | Annotation |
| --- | --- | --- | --- | --- | --- | --- |
|  |  |  | cell** | sup*** |  |  |
| 1 | MMOS7_11640 | Sec | 0 | 15.4 | 0 | Carbonic anhydrase |
| 2 | MMOS7_10670 | Sec | 0 | 14.4 | 0 | Hypothetical protein |
| 3 | MMOS7_09490 | Sec | 0 | 10.8 | 0 | S-layer protein precursor |
| 4 | MMOS7_11590 | Tat | 0 | 6.6 | 0 | [NiFe] hydrogenase small subunit |
| 5 | MMOS7_15050 | None | 0 | 5.3 | 0 | Hypothetical protein |
| 6 | MMOS7_15070 | None | 0 | 5.3 | 0 | Hypothetical protein |
| 7 | MMOS7_06450 | Sec | 0 | 3.1 | 0 | Hypothetical protein |
| 8 | MMOS7_13320 | Sec | 0 | 3 | 0 | Putative amino acid ABC transporter periplasmic binding protein |
| 9 | MMOS7_15990 | Sec | 0 | 2.7 | 0 | Hypothetical protein |
| 10 | MMOS7_12890 | Sec | 0 | 1.8 | 0 | Putative iron compound ABC transporter periplasmic binding protein |
| 11 | MMOS7_18230 | None | 0 | 1.5 | 0 | 30S ribosomal protein S27e |
| 12 | MMOS7_15450 | Sec | 0 | 1.3 | 0 | Hypothetical protein |
| 13 | MMOS7_02250 | Sec | 0 | 1.2 | 0 | Hypothetical protein |
| 14 | MMOS7_11600 | None | 0.1 | 4.9 | 2.3 | [NiFe] hydrogenase large subunit |
| 15 | MMOS7_07380 | Sec | 0.2 | 9.0 | 3.4 | Hypothetical protein |
| 16 | MMOS7_09720 | Sec | 0.1 | 2.0 | 6.9 | Hypothetical protein |
| 17 | MMOS7_05210 | Sec | 0.8 | 11.1 | 10.2 | Putative iron compound ABC transporter periplasmic binding protein |
| 18 | MMOS7_14510 | None | 20.5 | 214.4 | 13.9 | Archaeal histone |
| 19 | MMOS7_03470 | Sec | 0.3 | 1.5 | 24.8 | Hypothetical protein |
| 20 | MMOS7_08460 | Sec | 0.2 | 1.4 | 25.2 | Hypothetical protein |

* Signal sequences were detected by the PRED-TAT software^16^.

** emPAI of a protein isolated from cells [emPAI(cell)] collected from a 5.5-ml culture.

*** emPAI of the corresponding protein isolated from a 800-ml supernatant [emPAI(sup)].

**** emPAI(cell) per ml, [emPAI(cell)]/5.5, was divided by emPAI(sup) per ml, [emPAI(sup)]/800. This value, relative protein abundance (cell/sup), represents an estimate of the relative amount of protein in cells vs supernatant. The top 20 proteins with the lowest “relative protein abundance” values are shown.

Three proteins encoded in the MIC island are shaded.

**Supplementary figures**

**
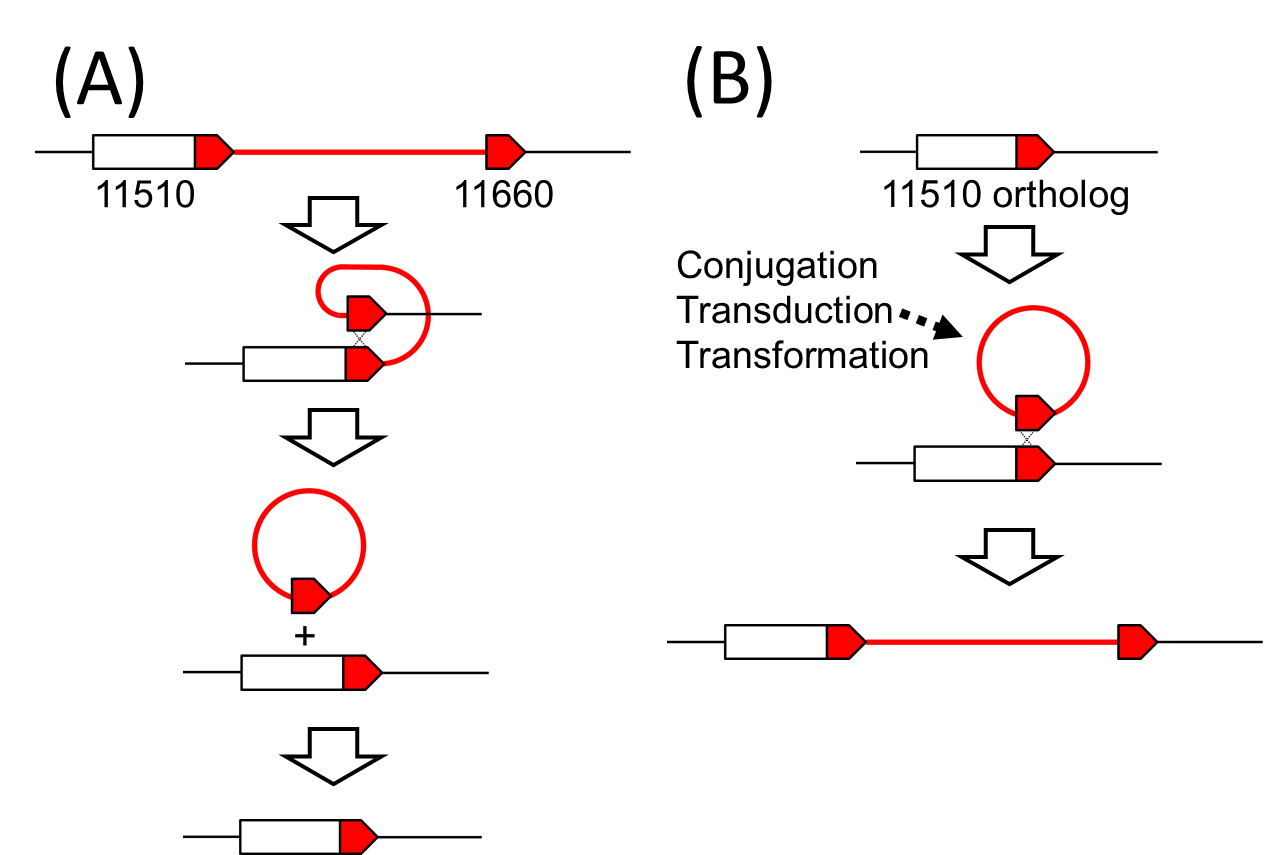
**

**Figure S1.** Excision of the MIC island from the chromosome of strains OS7 and KA1, and spread of the MIC island in local populations. Two red polygons represent directly repeated sequences flanking the MIC island, which is presented as a red line. **(A)** A homologous recombination event excises the MIC island as a circular DNA harboring one of the repeated sequence. Cell division after the excision event yields cells free from the MIC island.

**(B)** When the circular form of the MIC island DNA is introduced into a cell harboring a *MMOS7_11510*-like sequence (methanogenesis marker protein 1), the MIC island can be integrated into its chromosome, converting the cell from a MIC-negative to a MIC-positive phenotype.


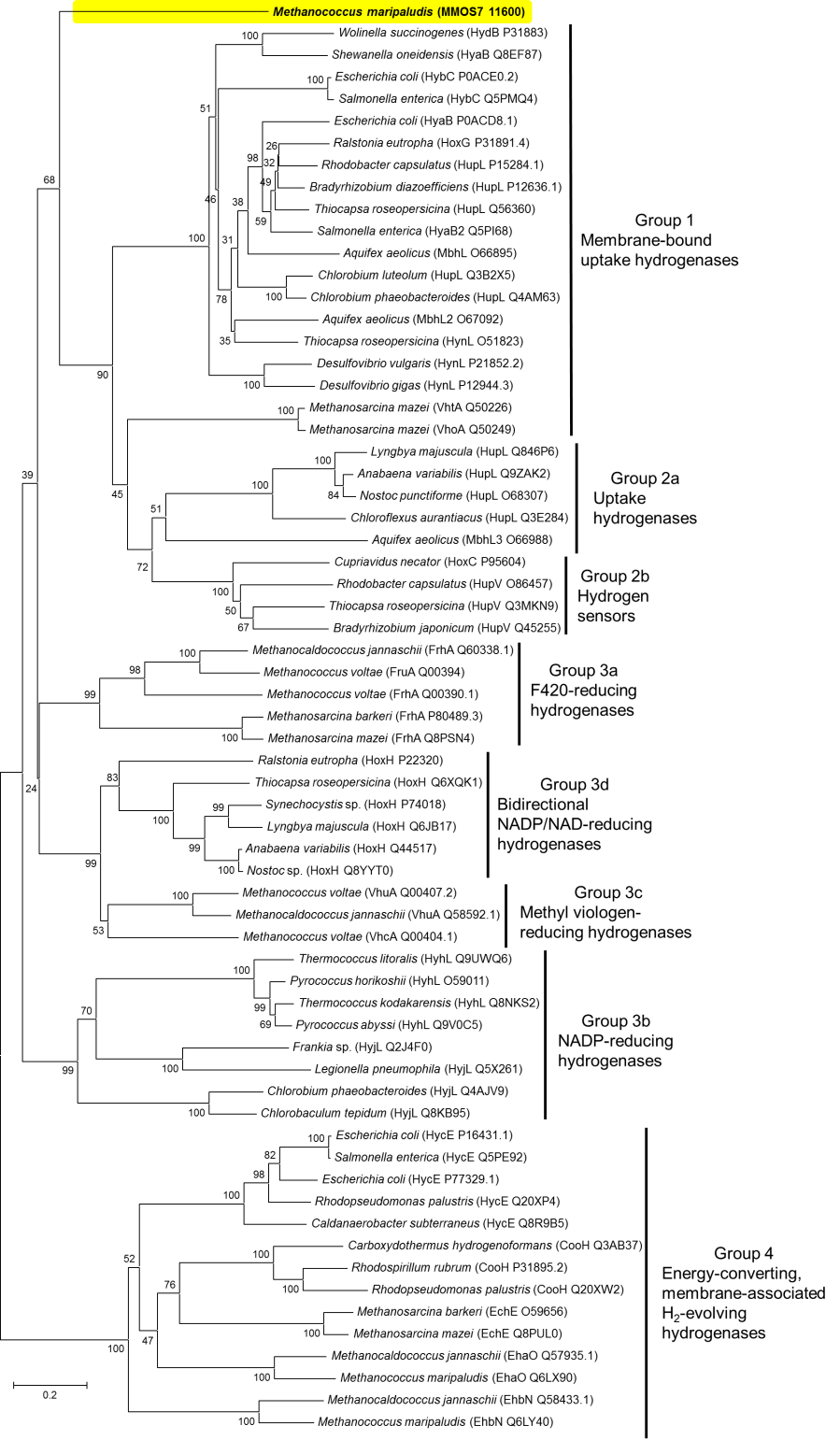


**Figure S2.** Phylogenetic tree of large subunits of [NiFe] hydrogenases. The amino acid sequence of MMOS7_11660 was compared to those of [NiFe] hydrogenases from diverse prokaryotes cited in a review^17^. A phylogenetic tree was constructed using the neighbor-joining method with MEGA 6.0 tools. Numbers at the tree nodes represent percentage bootstrap values after 1,000 replicates. For each [NiFe] hydrogenase, the scientific name of organisms, protein names, and accession numbers are given. Protein MMOS7_11600 is highlighted in yellow.


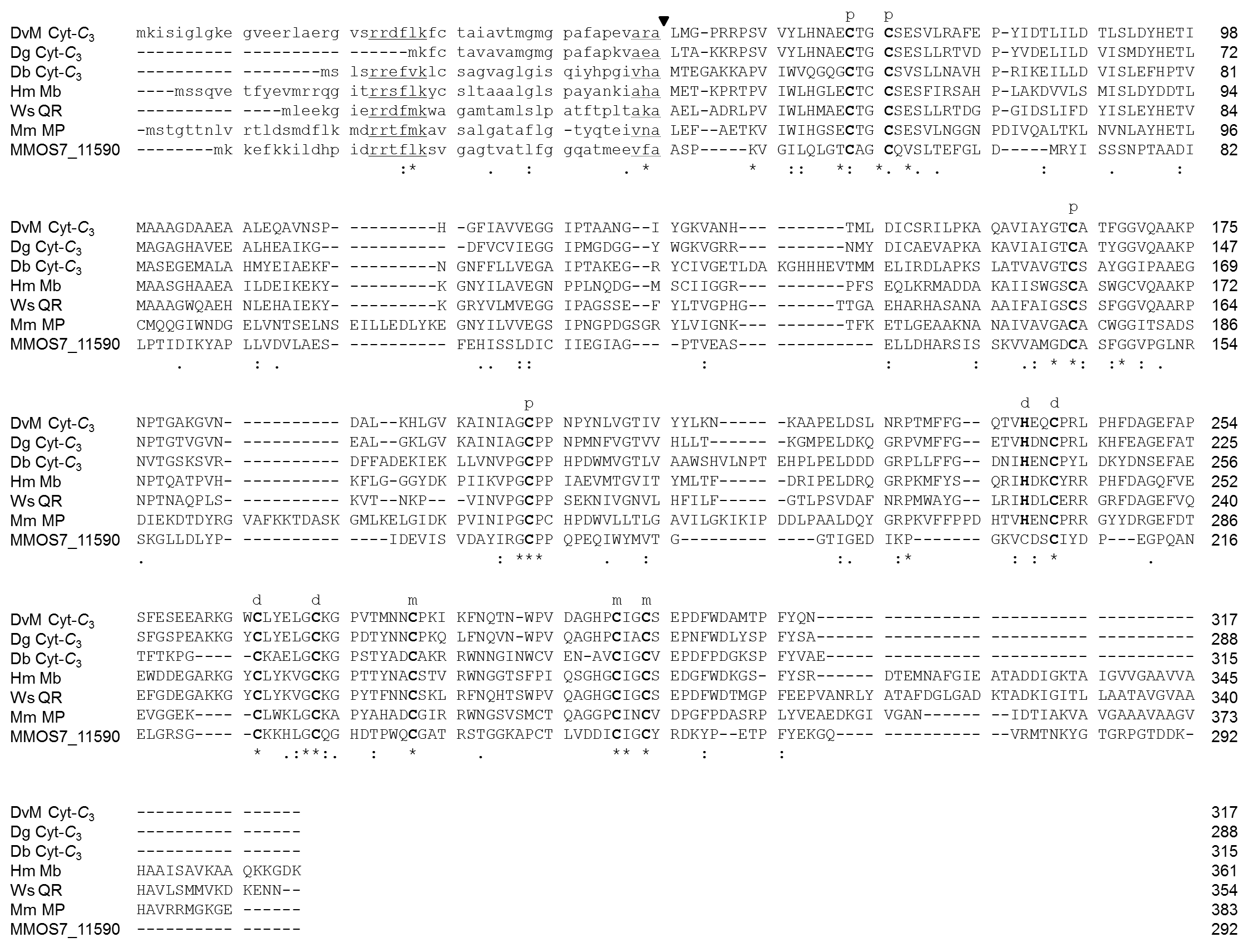


**Figure S3.** Multiple sequence alignment of amino acid sequences of [NiFe] hydrogenase small subunits. The amino acid sequence of MMOS7_11590 was compared to those of small subunits of the following [NiFe] hydrogenases cited in a review^17^ (abbreviation of organism name, accession number): *Desulfovibrio vulgaris* Miyazaki (Dv, P21853), *Desulfovibrio gigas* (Dg, P12943), *Desulfovibrio baculatum* (Db, P13063), *Hydrogenovibrio marinus* (Hm, BAK19333), *Wolinella succinogenes* (Ws, P31884), and *Methanosarcina mazei* (Mm, CAA58113). Signal sequences are shown by lowercase letters, and the cleavage site is indicated by an inverted triangle. The Tat motif (RRxFxK) and signal peptidase AxA recognition motif in the signal sequences are both underlined. Letters, "p", "m", and "d" above the aligned sequences indicate residues coordinating the proximal [4Fe-4S] cluster, mesial [3Fe-4S] cluster, and distal [4Fe-4S] cluster, respectively. These residues are highlighted in bold. An asterisk (*), colon (:), and period (.) indicate identical amino acids, conserved amino acids, and semi-conserved amino acids, respectively. Hyphens (-) indicate gaps introduced during multiple sequence alignment.


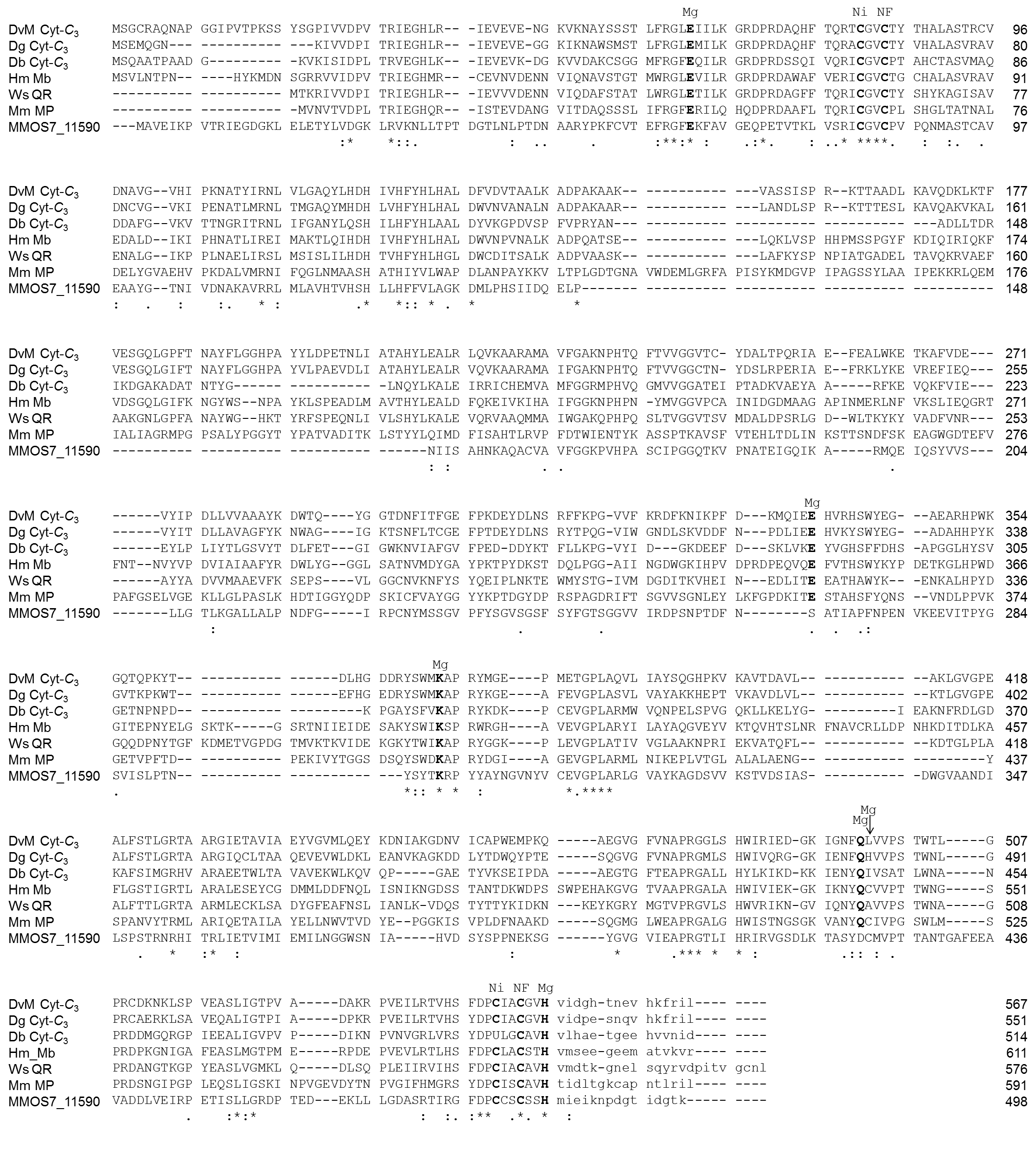
**Figure S4.** Multiple sequence alignment of amino acid sequences of [NiFe] hydrogenase large subunits. The amino acid sequence of MMOS7_11600 was compared to those of the large subunits of the same [NiFe] hydrogenases as presented in Figure S3. The accession numbers for the large subunit proteins are: Dv, P21852; Dg, P12944; Db, ACU90057; Hm, BAK19334; Ws, P31883; and Mm, CAA58114. Letters "Mg" above the aligned sequences indicate residues coordinating the Mg^2+^ center, while letters "Ni" and "NF" indicate residues coordinating Ni and Ni-Fe in the [NiFe] active site, respectively. These residues are highlighted in bold. The C-terminal amino acid sequences, which are expected to be removed by a hydrogenase maturation protease, are shown as lowercase letters.

**(A)**

*MCBB_1249* ATGGTGCAAAAGAAAAAGGGAACAAAAATTGTCATTCTTGGATCAGCAGA

*MMOS7_11510/20* ATAGATGTTTCAAGAGTAATTATTCCTAAAATGGAAATGTAC**GTG**TTGAT

** * * * * * * *

[11520 start codon]

*MCBB_1249* TTCTGGAAAAACAACCACAATAGAAAATCTCTTAAACCG**AAAAAAAGAAA**

*MMOS7_11510/20* AGAGATAGAATTTCACTCTGGATTAAAGATCGGATTCGAAG**AAACCTTGA**

* ** * * *** ** * * * *** *

*MCBB_1249* **AAATTACCAAAATTGAGTG**TAAAGGAACAACAGTAGCACTTGATTATGGA

*MMOS7_11510/20* **AAGTAAC**AAAAAT**TGA**GTGCAAGGGTACAACTGTTTCCCTCGATTATGGT

** * ** *********** ** ** ***** ** * ** ********

[11510 stop codon]

*MCBB_1249*  AACACGATAATCAACGGCCAAAGATTCCATATATTCGCCACTCCTGGCCA

*MMOS7_11520* AATACCATAATTAATGGCGAAAAAATCCATATTTTCGGCTCTCCAGGACA

** ** ***** ** *** *** * ******* **** * **** ** **

*MCBB_1249* AGAAAGATTCCAATTTATGCGTGAAATCCTTTCAAACGGGTTAGACGGTG

*MMOS7_11520* GGAAAGATTCAAATTCATGCGTGAAATCCTTTCAAACGGGTTAAATGGTG

********* **** *************************** * ****

*MCBB_1249* CAATCGTAGTTATAGACAACTCTGAAGGTGTTACAAACACAGATATAAAA

*MMOS7_11520*  CTATTGTAGTCATTGACAATTCAAGAGGAGTTACAGATACTGATATCCAA

* ** ***** ** ***** ** *** ****** * ** ***** **

*MCBB_1249* ATATTAGAAAATTTAAATTCAAGCAACGTTCCTTATGTAATATTCAGCAA

*MMOS7_11520* ATAATGGATAATTTAAACACCAATAACATTCCTTATGTAGTTTTTGCAAA

*** * ** ******** * * *** *********** * ** **

*MCBB_1249* TAAACAAGACATATCACCTGGAAAAATCGAATCAACACATATAAATCCTG

*MMOS7_11520* TAAACAGGATATATCTCCAGGAAATCCTGAATCTGAGTACATAGATTCTC

****** ** ***** ** ***** ***** * *** ** ***

*MCBB_1249* ATATTCCTGTAATTCCAACCACTGCAACAACTGGAGAAGGAATTCAGGAA

*MMOS7_11520*  ATATTCAAATAATCCCAACCATCGCAAAAGAAGGAGAAGGAATTCAGGAA

****** **** ******* **** * ******************

*MCBB_1249*  GGTTTGAACATTCTTTTAGATTTAATGGAAAATTAA

*MMOS7_11520*  GGATTAGAAATTCTTCTAGAATTAGTAGAAAACTAA

** ** * ****** **** *** * ***** ***

**(B)**

Ancestral *MMOS7_11510* **AAACCTTGAAAGTAACTTAAATTTAATTTAA**

**X**

Ancestral *NCBB_1249* **AAAAAGAAAAAATTACCAAAATTGAGTG**

Current *MMOS7_11520* **AAACCTTGAAAGTAACAAAAATTGAGTG**

**Figure S5.** Sequence alignment of *MCBB_1249* and *MMOS7_11520* is shown in **(A)**. The 74 bp 3′-end sequence of *MMOS7_11510* overlapping with the 5′-end of *MMOS7_11520* is underlined. *MMOS7_11520* may thus have been formed by the fusion between an ancestral *MMOS7_11510* gene and an ancestral *NCBB_1249* locus as shown in **(B)**. The ancestral *MMOS7_11510* sequence was deduced from that of *MMOS7_11660*.

*MCBB_1258*  ACTGAACGTAAAAAGATGGAAACCGCCTTGTCATGGGAAGTTTCAATTAA

*MMOS7_11650* ACTGAGCGTAAAAAGATGGAAATGGCATTGTCATGGGAAATGGCAATTAA

***** **************** ** ************ * *******

*MCBB_1258*  CAATGCTTTGGCTAAATTATCAAGAAATCTCCTGTCTCAAGCCTCAATTG

*MMOS7_11650* TAATGCTTTGGCTAAATTATCAAAAAAACTCCTCTCTCAAGCCTCAATTG

********************** *** ***** ****************

*MCBB_1258* ATGATATTTCATATCTTGTTTTAGAAC**ATGCCAAAGAT**CTAACCTGCAGC

*MMOS7_11650/60* AGGATATCACATATCTTGTTTTAAAAT**ATGCCAAATAA**TGCAAAATTAAA

* ***** ************** ** ******** * * *

[Start of 11660] [Stop of 11650]

*MCBB_1258*  CAATACGGTTTTGTTGGATATATAAATCCTAAAACAGGTTATCTAATGGT

*MMOS7_11660* TGCAAAATTAAATCTTAAAAAAGACATTGAAACAGTTAAAGAAATATTAA

* * * * * * * ** ** * **

**Figure S6.**  Alignment of the partial sequences of *MCBB_1258* and *MMOS7_11650*. The homology of *MMOS7_11650* extends to only the 5′-half of *MCBB_1258*. The last 11 nucleotides, ATGCCAAATAA (bold and underlined), of *MMOS7_11650* overlap *MMOS7_11660*. Since the same ATGCCAAATAA sequence was found in *MMOS7_11510* [see Figure 3 (B)], the current *MMOS7_11650 - MMOS7_11660* structure was expected to have been formed by fusion of the 5′-half of *NCBB_1258-*like sequence with *MMOS7_11660*.

**
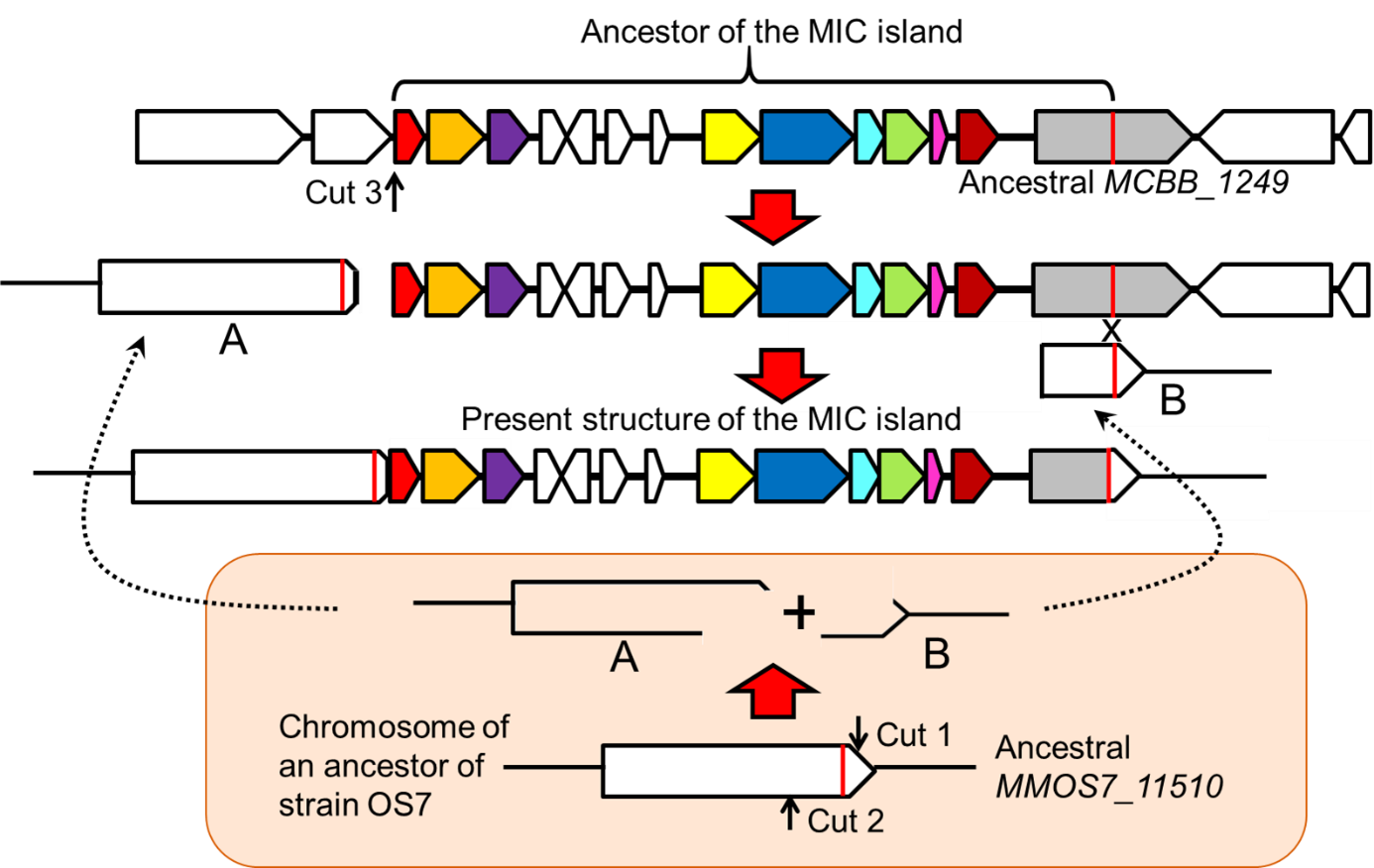
**

**Figure S7.**  Model of the formation of the present MIC island structure in strains OS7/KA1. We assume that an ancestral *MMOS7_11510,* which is a ubiquitous gene in methanogens, had been cleaved at approximately 15 bp upstream of the gene end in one strand (cut 1), and at > 234 bp upstream of the gene end in the other strand (cut 2) producing staggered ends which could be filled by the action of DNA polymerase. Thus, the ancestral *MMOS7_11510* locus had been rearranged into fragments A and B. We also assume that the structure of an ancestral MIC island is better-conserved in the gene cluster *MCBB_1249 – MCBB_1258* of strain Buetzberg. It is not clear whether the ancestors of the four genes (*MMOS7_11550 – MMOS7_11580*) existed in the ancestral MIC island. We further assume that the ancestral MIC island had been cleaved at approximately 100 bp downstream of the 5′-end of the ancestral *MCBB_1249* (cut 3), and the cleaved ancestral *MCBB_1249* had been joined to the 3′ end of fragment A of the ancestral *MMOS7_11510*. Subsequently, the ancestral *MCBB_1258* and fragment B recombined at the homologous ATGCCAAA sequence (see Figure S6), which is shown in red lines in both the ancestral *MMOS7_11510* and *MCBB_1249*.

**Reference**

1. Argyle, J. L., Tumbula, D. L. & Leigh, J. A. Neomycin resistance as a selectable marker in *Methanococcus maripaludis*. *Appl. Environ. Microbiol*. **62**, 4233-4237 (1996).
2. Ichikawa, N. *et al.* Genome sequence of *Kitasatospora setae* NBRC 14216^T^: an evolutionary snapshot of the family *Streptomycetaceae.* *DNA Res.* **17**, 393–406 (2010).
3. Masai, E. *et al.* Complete genome sequence of *Sphingobium* sp. strain SYK-6, a degrader of lignin-derived biaryls and monoaryls. *J. Bacteriol.* **194**, 534–535 (2012).
4. Takarada, H. *et al.* Complete genome sequence of the soil actinomycete *Kocuria rhizophila*. *J. Bacteriol.* **190**, 4139–4146 (2008).
5. Besemer, J., Lomsadze, A., & Borodovsky, M. GeneMarkS: a self-training method for prediction of gene starts in microbial genomes. Implications for finding sequence motifs in regulatory regions. *Nucleic Acids Res.* **29**, 2607–2618 (2001).
6. Delcher, A. L., Harmon, D., Kasif, S., White, O., & Salzberg, S. L. Improved microbial gene identification with GLIMMER. *Nucleic Acids Res.* **27**, 4636–4641 (1999).
7. Delcher, A. L., Bratke, K. A., Powers, E. C., & Salzberg, S. L. Identifying bacterial genes and endosymbiont DNA with Glimmer. *Bioinformatics* **23**, 673–679 (2007).
8. Altschul, S. F., Gish, W., Miller, W., Myers, E. W., & Lipman, D. J. Basic local alignment search tool. *J. Mol. Biol.* **215**, 403–410 (1990).
9. Bendtsen, J. D., Nielsen, H., von Heijne, G., & Brunak, S. Improved prediction of signal peptides: SignalP 3.0. *J. Mol. Biol.* **340**, 783–795 (2004).
10. Bendtsen, J. D., Nielsen, H., Widdick, D., Palmer, T., & Brunak, S. Prediction of twin-arginine signal peptides. BMC Bioinformatics **6**, 167 (2005).
11. Grifﬁths-Jones, S., *et al.* Rfam: annotating non-coding RNAs in complete genomes. *Nucleic Acids Res.* **33**, D121–D124 (2005).
12. Lowe, T. M., & Eddy, S. R. tRNAscan-SE: a program for improved detection of transfer RNA genes in genomic sequence. *Nucleic Acids Res.* **25**, 955–964 (1997).
13. Tamura, K., Stecher, G., Peterson, D., Filipski, A., & Kumar, S. MEGA6: molecular evolutionary genetics analysis version 6.0. *Mol. Biol. Evol.* **30**, 2725–2729 (2013).
14. Yamazaki, S., *et al.* Proteome analysis of an aerobic hyperthermophilic crenarchaeon, *Aeropyrum pernix* K1. *Mol. Cell. Proteomics* **5**, 811–823 (2006).
15. Ishihama, Y. *et al*. Exponentially modified protein abundance index (emPAI) for estimation of absolute protein amount in proteomics by the number of sequenced peptides per protein. Mol. Cell. Proteomics 4, 1265-1272 (2005).
16. Bagos, P. G., Nikolaou, E. P., Liakopoulos, T. D., & Tsirigos, K. D. Combined prediction of Tat and Sec signal peptides with hidden Markov models. *Bioinformatics* **26**, 2811–2817 (2010).
17. Yagi, T., & Higuchi, Y. Studies on hydrogenase. *Proc. Jpn. Acad. Ser.* B **89**, 16–33 (2013).
